# Guidelines for the diagnosis and treatment of neurally mediated syncope in children and adolescents (revised 2024)

**DOI:** 10.1007/s12519-024-00819-w

**Published:** 2024-08-07

**Authors:** Cheng Wang, Ying Liao, Shuo Wang, Hong Tian, Min Huang, Xiang-Yu Dong, Lin Shi, Ya-Qi Li, Jing-Hui Sun, Jun-Bao Du, Hong-Fang Jin, Chinese Pediatric Cardiology Society, Chinese Pediatric Society, Chinese Medical Association; Committee of Pediatric Syncope, College of Pediatricians, Chinese Medical Doctor Association; Pediatric Cardiology Society, Beijing Pediatric Society, Beijing Medical Association; Committee of Pediatric Cardiology, College of Cardiovascular Physicians, Chinese Medical Doctor Association, Jin-Dou An, Xin-Jiang An, Jie Chen, Li-Qing Chen, Ming-Wu Chen, Shu-Qin Chen, Qi Chen, Yong-Hong Chen, Sun Chen, Zhi Chen, Adolphus Kai-tung Chau, Mao-Ping Chu, Hui-Ying Cui, Xiang-Yu Dong, Jun-Bao Du, Shu-Xu Du, Zhong-Dong Du, Hong-Yu Duan, Jun-Kai Duan, Lin Feng, Li-Jun Fu, Fang Gao, Lu Gao, Wei Gao, Fang-Qi Gong, Li Gu, Hong Gu, Yong-Hao Gui, Zhen-Hui Han, Bo Han, Ling Han, Bing He, Xue-Hua He, Zhi-Xu He, Xiu-Fen Hu, Yao-Fei Hu, Yi-Min Hua, Guo-Ying Huang, Hui-Tao Huang, Min Huang, Ping Huang, Xing-Yuan Huang, Yu-Juan Huang, Shou-Yuan Jiang, Hong-Fang Jin, Mei Jin, Yan-Zhe Lei, Bo Li, Fen Li, Li Li, Tao Li, Xiao-Ming Li, Xiao-Hui Li, Yan Li, Yun Li, Zi-Pu Li, Yong-Mei Liang, Ying Liao, Fang Liu, Wei Liu, Xiao-Yan Liu, Ya-Li Liu, Hui-Ling Lu, Hai-Tao Lv, Tie-Wei Lv, Lu-Yi Ma, Bao-Quan Pan, Xiang-Bin Pan, Si-Lin Pan, Yu-Sheng Pang, Hua Peng, Jin-Hua Piao, Ming-Yang Qian, Wei Qian, Yu-Ming Qin, Jie Shen, Lin Shi, Guo-Dong Song, Jing-Hui Sun, Hong Tian, Jie Tian, Cheng Wang, Cui-Ling Wang, Hong Wang, Lei Wang, Li-Hong Wang, Lin Wang, Qin Wang, Shu-Shui Wang, Wen-Di Wang, Xiao-Ning Wang, Yi-Biao Wang, Jian-Xin Wu, Rong-Zhou Wu, Yu-Rong Wu, Kun Xia, Ting-Ting Xiao, Yan-Yan Xiao, Li-Jian Xie, Yu-Mei Xie, Chun-Hong Xie, Yan-Lin Xing, Zhen-Yu Xiong, Bao-Yuan Xu, Yi Xu, Hui Yan, Jian-Ping Yang, Shi-Wei Yang, Qi-Jian Yi, Xia Yu, Xian-Yi Yu, Yue Yuan, Du-Fei Zhang, Hong-Yan Zhang, Hui-Li Zhang, Kun Zhang, Li Zhang, Ming-Ming Zhang, Qing-You Zhang, Xi Zhang, Yan-Min Zhang, Yong Zhang, Zhi-Wei Zhang, Cui-Fen Zhao, Bin Zhou, Kai-Yu Zhou, Hua Zhu, Sheng-Dong Zhu, Jian-Xin Zhuang

**Affiliations:** 1 Department of Pediatric Cardiovasoloy, Children’s Medical Center, The Second Xiangya Hospital, Central South University, Changsha, 410011 China; 2 Department of Pediatrics, Peking University First Hospital, Beijing, 100034 China; 3 Department of Pediatrics, Xiangya Hospital, Central South University, Changsha, 410008 China; 4 Department of Pediatric Cardiology, Children’s Hospital of Fudan University, Shanghai, 201102 China; 5 Department of Pediatric Cardiology, Shanghai Children’s Hospital, Shanghai, 201102 China; 6 Department of Pediatrics, Lanzhou University Second Hospital, Lanzhou, 730020 China; 7 Department of Pediatric Cardiology, Capital Institute of Pediatrics, Beijing, 100020 China; 8 Department of Pediatrics, Jilin University First Hospital, Changchun, 130021 China; 9 State Key Laboratory of Vascular Homeostasis and Remodeling, Peking University, Beijing, 100191 China

**Keywords:** Adolescents, Children, Diagnosis, Neurally mediated syncope, Treatment

## Abstract

**Background:**

Significant progress has been made in the diagnosis and treatment of pediatric syncope since the publication of the “2018 Chinese Pediatric Cardiology Society (CPCS) guideline for diagnosis and treatment of syncope in children and adolescents” (“2018 Edition Guidelines”). Therefore, we have revised and updated it to assist pediatricians in effectively managing children with syncope.

**Data sources:**

According to the “2018 Edition Guidelines”, the expert groups collected clinical evidence, evaluated preliminary recommendations, and then organized open-ended discussions to form the recommendations. This guideline was developed by reviewing the literature and studies in databases including PubMed, Cochrane, EMBASE, China Biomedical Database, and Chinese Journal Full-text Database up to April 2024. Search terms included “syncope”, “children”, “adolescents”, “diagnosis”, and “treatment.”

**Results:**

The guidelines were based on the latest global research progress and were evidence-based. The classification of syncope etiology, diagnostic procedures, postural tests, such as the active standing test, head-up tilt test, and active sitting test, clinical diagnosis, and individualized treatment for neurally mediated syncope in pediatric population were included.

**Conclusions:**

The guidelines were updated based on the latest literature. The concepts of sitting tachycardia syndrome and sitting hypertension were introduced and the comorbidities of neurally mediated syncope were emphasized. Some biomarkers used for individualized treatment were underlined. Specific suggestions were put forward for non-pharmacological therapies as well as the follow-up process. The new guidelines will provide comprehensive guidance and reference for the diagnosis and treatment of neurally mediated syncope in children and adolescents.

## Introduction

Syncope is a transient loss of consciousness (TLOC) and inability to maintain the posture caused by transient global cerebral hypoperfusion. It is characterized by a rapid onset, short duration, and spontaneous complete recovery [1–4] (I; C). Syncope, a common disorder in children and adolescents, has an incidence of 17.37% [5], with a higher occurrence in girls than in boys [6] (IIa; B). It accounts for 1%–2% of visits to the emergency department [1]. The pathogenesis, etiology, diagnosis, and treatment of pediatric syncope differ from those in adults. Recurrent syncope can seriously impact the physical and mental health, learning abilities, and quality of life of affected children [7, 8] (IIa; A). In some cases, syncope with cardiogenic causes can even pose a risk of sudden death. From 2015 to 2018, the Heart Rhythm Society, the American College of Cardiology/American Heart Association Task Force on Clinical Practice Guidelines, the Canadian Cardiovascular Society, the Canadian Pediatric Cardiology Association, and the European Society of Cardiology successively published guidelines or society position statements on the diagnosis and management of syncope and related syndromes [2, 9–11] (I; C). In 2018, professional societies including the Chinese Pediatric Cardiology Society, Chinese Pediatric Society, Chinese Medical Association; Committee of Pediatric Syncope, College of Pediatricians, Chinese Medical Doctor Association; Pediatric Cardiology Society, Beijing Pediatric Society, Beijing Medical Association; Committee of Pediatric Cardiology, College of Cardiovascular Physicians, Chinese Medical Doctor Association released the "2018 Chinese Pediatric Cardiology Society (CPCS) guidelines for diagnosis and treatment of syncope in children and adolescents" (hereafter referred to as the "2018 Edition Guidelines") [1] to promote the standardized diagnosis and treatment of syncope in children and adolescents. In recent years, significant progress has been made in the clinical diagnosis and treatment of syncope, especially neurally mediated syncope (NMS) in children and adolescents, including the etiological classification of syncope, diagnostic procedures, posture tests, such as the active standing test, the head-up tilt test (HUTT), as well as the active sitting test, clinical diagnosis, and individualized treatment [12–25]. The “Guidelines for the diagnosis and treatment of neurally-mediated syncope in children and adolescents (revised 2024)” were developed based on the "2018 Edition Guidelines" and followed the latest global research advances. However, more large-scale, multi-center, randomized controlled clinical studies are needed to provide a higher level of evidence for the treatment of NMS in children and adolescents [26].

## Classes of recommendations and levels of evidence to manage pediatric syncope

The classes of recommendations and levels of evidence for the management of pediatric syncope were weighed and graded based on predefined scales (Tables 1, 2) [1, 2] (I; C).

**Table 1:** Classes of recommendations for the management of pediatric syncope

Classes of recommendations	Definition	Suggested wording to use
Class I	Evidence and/or general agreement that the given treatment or procedure is beneficial, useful and effective	Is recommended/indicated
Class II	Conflicting evidence and/or a divergence of opinion about the usefulness/efficacy of the given treatment or procedure	
Class IIa	Weight of evidence/opinion favors the usefulness/efficacy	Should be considered
Class IIb	Usefulness/efficacy is less well established by evidence/opinion	May be considered
Class III	Evidence or general agreement that the given treatment or procedure is not useful/effective and in some cases may be harmful	Is not recommended

**Table 2:** Levels of evidence for the management of pediatric syncope

Level of evidence	Source of evidence
A	Data derived from multiple randomized clinical trials or meta-analyses
B	Data derived from a single randomized clinical trial or large non-randomized studies
C	Consensus of opinion of experts and/or small studies, retrospective studies, registries

## The underlying diseases of syncope in children and adolescents

The causes of syncope in children and adolescents include NMS, cardiac syncope (CS), and unexplained syncope (UPS) [1]. NMS is the most common cause accounting for 70%–80%, CS accounts for 2%–3%, and UPS accounts for about 20% [7] (I; C). The causes of syncope in children and adolescents are classified and listed in Table 3.

**Table 3:** Classification of syncope in children and adolescents

Classes	Underlying diseases and associated syndrome
Neurally mediated syncope	Vasovagal syncope Vasoinhibitory type Cardioinhibitory type Mixed type Postural orthostatic tachycardia syndrome Orthostatic hypotension Orthostatic hypertension Sitting tachycardia syndrome Sitting hypertension
	Situational syncope
	Carotid sinus syndrome
Cardiac syncope	Arrhythmia Structural cardiac diseases, including cardiomyopathy, etc.
Unexplained syncope	

NMS in children and adolescents is characterized by syncopal attacks caused by abnormal reflex regulation or dysfunction of the autonomic nervous system, mainly referring to vasovagal syncope (VVS) and postural orthostatic tachycardia syndrome (POTS), which accounts for about 95% of NMS [4, 27–30] (IIa; B). Breath-holding spells in infancy may be a specific type of NMS [31, 32] (I; C). In children and adolescents, NMS is often associated with comorbidities [33, 34]. Nearly 30%–40% of children and adolescents with VVS and/or POTS generally also have allergic diseases [35] (IIa; B), and some may also be associated with coagulation disorders [36], but patent foramen ovale may not increase the risk of pediatric syncope [37] (IIa; B). Other common comorbidities include migraines, mental disorders, sleeping disorders, hyperventilation syndrome, chronic fatigue syndrome, joint hypermobility syndrome, and gastrointestinal disorders. Comorbidities may increase the complexity of diagnosis and treatment [38] (IIa; B).

CS is mainly caused by abnormal structure or rhythm of the heart. Although CS is a relatively rare cause of pediatric syncope, it is associated with a high risk of sudden death and needs to be diagnosed as early as possible [39] (IIa; B).

Other causes of TLOC [7, 40] (IIa; A), including epileptic seizures, metabolic disorders (e.g., hypoglycemia and hypoxemia), poisoning, and psychological pseudosyncope (PPS) [41–43] (IIa; B), can sometimes be misdiagnosed as syncope. These disorders do not produce transient cerebral hypoperfusion, and thus, should be strictly differentiated from syncope.

## Diagnosis

### Diagnostic procedures for pediatric syncope

**Fig. 1:**
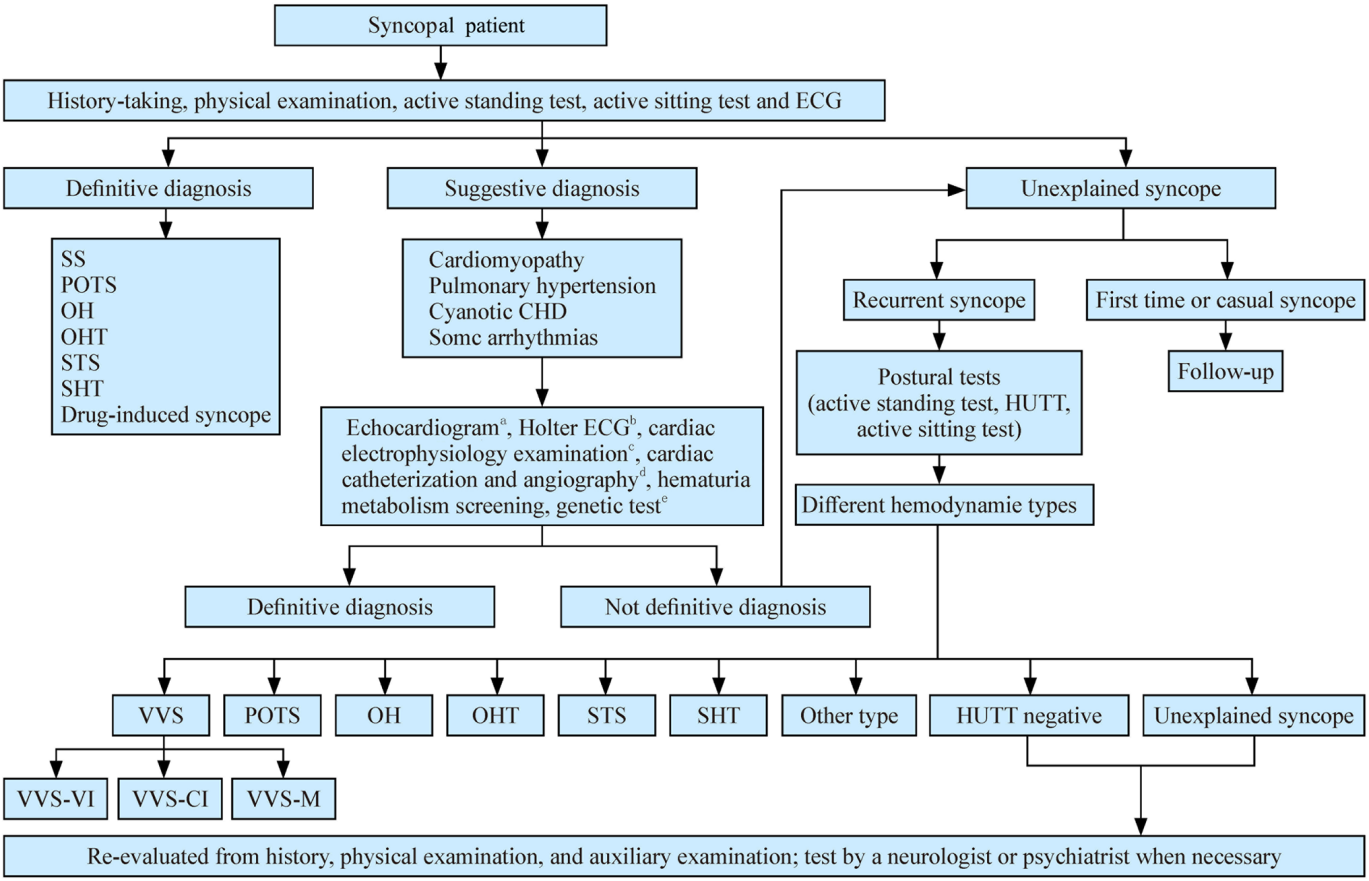
Diagnostic procedure for pediatric syncope. *ECG* electrocardiogram, *SS* situational syncope, *POTS* postural orthostatic tachycardia syndrome, *OH* orthostatic hypotension, *OHT* orthostatic hypertension, *STS* sitting tachycardia syndrome, *SHT* sitting hypertension, *CHD* congenital heart disease, *HUTT* head-up tilt test, *VVS* vasovagal syncope, *VVS-VI* vasovagal syncope vasoinhibitory type, *VVS-CI* vasovagal syncope cardioinhibitory type, *VVS-M* vasovagal syncope mixed type. ^a^For children with normal physical examination and normal routine ECG findings, ECG is generally not helpful in determining possible reasons. For children with possible structural heart defects after medical history, physical examination, and routine ECG. ECG is a screening tool to detect abnormal cardiac structures or functions; ^b^Holter ECG is a common test to determine the cause of syncope. However, because syncope is unpredictable, regular monitoring for only 24 hours makes it difficult to confirm or thoroughly rule out the association between arrhythmia and syncope. When diagnosing the cause of syncope, comprehensive judgment should be made together with history-taking and other tests. The possible reasons include asymptomatic sinus bradycardia, atrioventricular block, and endless supraventricular or ventricular tachycardia. For children with recurrent syncope, Holter ECG and ILR are important for diagnosis and differential diagnosis. For children with syncope induced by sports and emotions, an exercise test should be performed to detect potential arrhythmias. During exercise trials, first aid is prepared as a precaution; ^c^for patients with suspected sick sinus syndrome, atrioventricular conduction abnormalities, and/or all ventricular and supraventricular arrhythmias, the diagnosis can be confirmed by cardiac electrophysiological examination when necessary; ^d^for patients with suspected pulmonary hypertension or coronary heart disease, although ECG cannot clarify the diagnosis, cardiac catheterization and angiocardiography may be considered; ^e^for patients with suspected hereditary disease, such as ion channel diseases, cardiomyopathy, or genetic metabolic diseases, and for those with a family history of genetic heart disease or sudden death, the diagnosis may be confirmed by metabolic disorder screening and/or genetic tests

As shown in Fig. 1, the diagnostic procedures comprise three steps [44] (IIa; B). In the first step, after medical history taking, physical examination, supine and upright blood pressure (BP) measurement, and supine and upright electrocardiogram (ECG) recording, the patients can be divided into three groups: definite diagnosis, suggestive diagnosis, and UPS [1].

#### Definitive diagnosis

POTS, orthostatic hypotension (OH), orthostatic hypertension (OHT) [45, 46] (IIa; A), sitting tachycardia syndrome (STS), and sitting hypertension (SHT) [47, 48] (IIa; B) can be indicated by the history of symptoms of orthostatic intolerance (OI) or sitting intolerance. A diagnosis can be confirmed when patients with typical symptoms have a normal ECG and a positive result in an active standing test, HUTT, or active sitting test [49–56] (IIa; B). Situational syncope (SS) is defined as syncope in specific situations, such as micturition, defecation, bathing, swallowing, cough, post-dinner, singing, teeth brushing, and hair grooming [57, 58] (IIa; B). Drug-induced syncope (determined through medication history) can be diagnosed by typical history.

#### Suggestive diagnosis

The patients with suggestive diagnosis after the first step, as seen in Fig. 1, need further laboratory investigations in step 2. In step 2, cardiomyopathy, pulmonary hypertension, cyanotic congenital heart disease, and some types of arrhythmias can be suspected according to the clues from history, physical examination, and ECG findings. For example, onset in infancy or early childhood, exercise-induced events [59] (IIa; B), family history of genetic heart disease or sudden death, and abnormal ECG findings can suggest CS, with the strongest clues provided by exercise-induced syncope and abnormal ECG findings [60, 61]. For such patients, further examinations are needed to ensure the diagnosis, such as repetitive ECG, Holter ECG, implanted loop recorder (ILR) [62] (IIa; B), exercise test, intracardial electrophysiology, angiography, cardiovascular imaging, screening of metabolic disorder or genetic tests, based on the specific situation [46, 63] (IIa; B).

#### Unexplained diagnosis

In step 3, for patients whose diagnosis cannot be confirmed or indicated by detailed medical history, physical examination, supine BP, and ECG measurements [48] (IIa; A), if the symptoms occur more than once and the characteristics of the attacks suggest NMS, postural tests such as the active standing test, HUTT [9, 64–66] (IIa; B), and active sitting test [47, 48] (IIa; A) can be performed. These tests may help in diagnosing NMS and determining its hemodynamic type.

If a definite diagnosis cannot be achieved even after the above steps are taken, a re-evaluation is needed. This includes history-retaking, physical re-examination, and further laboratory examinations. Moreover, assessment by associated specialists such as neurologists or psychiatrists should be considered when necessary.

### Methodology of postural tests

#### Active standing test

The active standing test can be performed to screen for underlying causes of OI in children. It has no absolute contraindications.

##### The protocol

First, the children are asked to lay supine for 10–30 minutes to obtain supine heart rate (HR), BP, and ECG recordings. Then, the children are told to actively stand for another 10 minutes, simultaneously monitoring the above parameters [65–67] (IIa; A).

##### Clinical comments

During the active standing test, the children should be closely observed to determine whether they have presyncope symptoms or even syncope, and the test can help diagnose POTS [66, 67], OH [66, 68], or OHT [66, 69] (IIa; B). Here, the presyncope consists of symptoms including dizziness, headache, chest tightness, palpitations, nausea, vomiting, pallor, cold sweats, blurred vision, hearing loss, abdominal pain, and variable degrees of altered consciousness without complete loss of consciousness. HR, BP, and ECG are monitored in the supine position. When the ECG electrode does not move, observations of OI, HR, BP, and ECG are made for 10 minutes after assuming an upright position. Changes in HR and ECG waveforms between supine and upright positions are compared. Significant changes in T-wave morphology of decubitus and upright ECG help judge autonomic nervous function [70, 71] (IIa; B). In patients with clinical manifestations of OI but with a negative active standing test result, the HUTT could be considered if they do not have contraindications.  Criteria for the positive response for POTS, OH and OHT refer to relevant section of “Criteria for the positive response of active standing test and head-up tilt test”.

#### Head-up tilt test

##### Indications and contraindications

Indications for the HUTT include (1) to clinically confirm a suspected NMS, which cannot be diagnosed by other tests [1, 72] (I; C) and (2) to make a differential diagnosis with pseudosyncope [42, 73] (IIa; B). Contraindications for the HUTT are (1) known aortic stenosis or left ventricular outflow tract obstruction; (2) severe mitral stenosis; (3) definite pulmonary hypertension or right ventricular outflow tract obstruction; (4) serious stenosis in the proximal coronary artery; (5) definite severe cerebrovascular diseases; and (6) syncope caused by definite arrhythmia [1] (I; C).

##### Preparation for the head-up tilt test

The test environment must be quiet with dim light, a comfortable room temperature, and no distractions [1] (I; C). Medicines such as nitroglycerin tablets for sublingual nitroglycerin-provoked HUTT, adrenaline, atropine, and first aid equipment, including an oxygen inhalation device and defibrillator, should be prepared.

The tilt table should have supported foot pedals, with fences on both sides and fixed belts for the chest and knee joints to avoid knee flexion and falling. The electric table board should be well-controlled and move smoothly so that the table can be tilted to a certain angle from the horizontal position and return to a horizontal position from any tilted angle [74] (IIa; B).

The operators should cautiously observe the details of the patient’s manifestations suggesting syncope or presyncope attacks and must be familiar with the rules to stop the test and rescue program of the HUTT [75] (I; C). The detailed procedure of the HUTT should be explained to children and their legal guardians/parents to relieve their anxiety and obtain their cooperation during the whole test [1] (I; C).

Informed consent: the HUTT has certain potential risks, such as a fainting attack or significant hemodynamic changes such as cardiac asystole [76–80] (IIa; B). Therefore, detailed instructions and risks should be described to children and their legal guardians/parents, and signed informed consent should be obtained before the test [81] (IIa; B).

Preparation of the pediatric patient: any vasoactive drugs should be discontinued for at least five half-lives. Food and beverages, such as coffee, that might affect autonomic nervous system functions should be avoided for at least 24 hours before the test. Children should fast for at least 4 hours before the test. The test should be performed in the morning [67, 82] (IIa; B) without the presence of unrelated persons [72].

##### Steps of the head-up tilt test

Basic HUTT (BHUT): children should first lie on the tilt bed in a horizontal position for around 10–30 minutes with fixed bands to avoid buckling of knee joints. HR, BP, and ECG recordings are taken during this period. Then, the bed is tilted upward at an angle of 60° and HR, BP, and ECG are simultaneously monitored. The test must be stopped, and the children should be placed in the supine position (from the upright position) when the children show a positive response (see section “Criteria for the positive response”) or have completed the whole 45 minutes process, or if the patient requires stopping for any reason [83, 84] (IIa; B).

Sublingual nitroglycerin-provoked HUTT (SNHUT): if no positive response has been developed during the BHUT, the children are suggested to undergo a SNHUT, in the same position for a further 20 minutes after sublingual administration of nitroglycerin at a dose of 4–6 μg/kg (maximum ≤ 300 μg) [85] (IIa; B). The endpoint of the test is a positive response or completion of the process. After the administration of the medicine as mentioned above, HR, BP, ECG, and clinical performance should be carefully recorded simultaneously [86, 87] (IIa; A).

##### Criteria for the positive response of active standing test and head-up tilt test

Vasovagal syncope: syncopal episodes or presyncope symptoms together with any of the following responses in the HUTT are considered positive responses: (1) systolic BP (SBP) ≤ 80 mmHg (1 mmHg = 0.133 kPa) or diastolic BP (DBP) ≤ 50 mmHg, or mean pressure decrease ≥ 25%; (2) HR < 75 bpm for children aged 4 to 6 years, < 65 bpm for children aged > 6 to 8 years, and < 60 bpm for children and adolescents > 8 years; (3) ECG showing sinus arrest or junctional escape rhythm; (4) atrioventricular block (II or III degree) or cardiac arrest ≥ 3 seconds. The responses are classified as VVS vasoinhibitory type (VVS-VI), VVS cardioinhibitory type (VVS-CI), or VVS mixed type (VVS-M). VVS-VI is characterized by a significant decrease in BP without an obvious decrease in HR, VVS-CI by a marked decrease in HR without a marked decrease in BP, and VVS-M by both an HR and BP decrease [1, 45, 88, 89] (IIa; A). Malignant VVS is characterized by cardiac arrest lasting more than 3 seconds during a syncope episode or the test [90, 91] (IIa; B).

Postural orthostatic tachycardia syndrome: the diagnosis of POTS is influenced by the circadian rhythm [92, 93] (IIa; B). It was reported that, during the active standing test for children, the difference in HR between supine and upright positions is more significant in the morning when compared with that in the afternoon or the evening [67] (IIa; B). Therefore, it is recommended to take a posture test in the morning (IIa; B). During the upright position of the active standing test or the initial 10 minutes of HUTT, a positive response for POTS is defined as a HR increase of ≥ 40 bpm or a maximum upright HR ≥ 130 bpm (in children aged 6 to 12 years old) or ≥ 125 bpm (in adolescents over 12 years older but under 18) [66, 87] (IIa; A). However, all positive responses should exclude significant BP decrease (where SBP decreases by > 20 mmHg and/or DBP decreases by > 10 mmHg).

Orthostatic hypotension: for classic OH, the supine BP is normal. During the initial 3 minutes of the active standing test or HUTT, SBP decreases at least by 20 mmHg and/or DBP decreases at least by 10 mmHg [1, 68] (I; C). The initial OH is defined by a transient BP decrease occurring within 15 seconds after standing.

Orthostatic hypertension: supine BP is normal and during the initial 3 minutes of the active standing test or HUTT, SBP increases by ≥ 20 mmHg and/or DBP increases by ≥ 25 mmHg (in children aged 6–12 years) or ≥ 20 mmHg (in adolescents aged 12–18 years) from supine to upright position; or upright BP is ≥ 130/90 mmHg (in children aged 6–12 years) or ≥ 140/90 mmHg (in adolescents aged 12–18 years) without an obvious change in HR [66, 94, 95] (IIa; B).

##### Characteristics of the head-up tilt test in children

The autonomic nervous system of children is continuously developing and maturing, rendering it more unstable compared to that of adults. As a result, the hemodynamic parameters of the HUTT change more rapidly and abruptly, leading to positive responses appearing quite early in children. Additionally, children tend to cooperate less during the test than adults. Therefore, it is important to closely monitor and obtain the cooperation of children as much as possible during the HUTT. In most cases, children with POTS are more likely to demonstrate symptoms of OI than those with OH [96] (IIa; B). ECG changes such as sinus arrhythmia and sinus bradycardia in HUTT can predict an increased probability of positive responses [77] (IIa; B). Abnormal T-wave patterns, T-wave apex to T-wave end interval dispersion (Tped), and prolongation of the QT interval during the HUTT are useful in identifying children with VVS, who are more susceptible to ventricular arrhythmias with a latent risk of long QT syndrome [97] (IIa; B).

##### Safety issues of the head-up tilt test

The HUTT should be performed following the indications and operation protocols to guarantee safety. The HUTT may induce syncope or presyncope symptoms leading to adverse events or complications such as arrhythmias [77], transient aphasia [78], convulsions [79], and fear [98] (IIa; B). However, the HUTT is generally considered safe when performed in accordance with the recommendations of the standard HUTT protocol and when syncope caused by organic heart diseases is fully excluded [72, 76, 81] (IIa; B). During the HUTT, long RR intervals > 2 seconds in ECGs are not rare. Returning the patient to the recumbent position in time can promote recovery in consciousness. For children aged 3–18 years, the HUTT demonstrates high specificity, and severe HUTT-related complications have been rarely reported in recent studies [76, 99, 100] (IIa; B).

#### Active sitting test

The operation is simple and relatively safe, serving as a preliminary screening tool to identify the basic etiology in children and adolescents presenting symptoms of sitting intolerance such as dizziness, blurred vision, headache, chest tightness, nausea, abdominal pain, numbness and sweating when sitting, without clear contraindications [47] (IIa; B).

##### The protocol

After the children empty their bladder, the active sitting test is carried out in a quiet and comfortable room. The children are first asked to be in a supine position for 10 minutes and then in a sitting position for 10 minutes. Changes in HR and BP during sitting, as well as whether dizziness, pale face, fatigue, blurred vision, chest tightness, palpitations, abdominal pain, nausea, vomiting, and other symptoms of sitting intolerance occur during the test are dynamically observed and recorded. If the children are unable to complete the test, the test is terminated prematurely. STS and SHT can be diagnosed by an active sitting test [47, 101] (IIa; B).

##### Criteria for the positive response of active sitting test

Sitting tachycardia syndrome: symptoms of sitting intolerance appear within 10 minutes after the change from a supine to a sitting position, and an increase in HR by ≥ 25 bpm after 3 minutes of sitting suggests STS [47, 102, 103] (IIa; B).

Sitting hypertension: symptoms of sitting intolerance occur within 10 minutes after the transition from a supine to a sitting position, and SBP elevation by ≥ 20 mmHg and/or DBP elevation by ≥ 20 mmHg within 3 minutes of sitting suggest SHT [47, 102, 103] (IIa; B).

### Diagnostic criteria of neurally mediated syncope and associated syndromes

A detailed medical history can provide valuable information for diagnosis [60, 104] (IIa; A), and adherence to diagnostic guidelines may facilitate in correctly diagnosing patients experiencing syncope while avoiding unnecessary medical tests [39, 105–107]. Syncope is one of the common clinical manifestations of NMS in children and adolescents, although some cases can start with non-syncope symptoms, such as dizziness [108], tightness or pain in the chest [109], sigh or hyperventilation [110], palpitations [111], and abdominal pain [112, 113] (IIa; B).

#### Vasovagal syncope

The diagnostic criteria of VVS are as follows: (1) in most patients, it is associated with predisposing factors, such as persistent standing or sudden changes in body position (body position quickly changing to an upright position from supine or sitting or squatting), emotional stress, and crowded or stuffy environment; (2) a history of syncope is confirmed; (3) it features a positive hemodynamic response during a HUTT (see section “Criteria for the positive response of active standing test and head-up tilt test”); and (4) other causes of TLOC should be excluded [1, 114, 115] (IIa; B).

#### Postural orthostatic tachycardia syndrome

The diagnostic criteria of POTS are as follows: (1) the course of the disease is usually > 1 month and is often associated with most of the above-mentioned inducing factors (as seen in VVS). There are also some risk factors such as high basal HR in a supine position (a 10 bpm increase in HR in a supine position indicates an increased risk of POTS by 1.58 times), low water intake (water intake of children < 800 mL/day increases the risk of POTS by 3.88 times), insufficient sleep duration (i.e., children sleeping < 8 hours/day results in a 5.91 times increase in the risk of POTS) [116] (IIa; A); (2) OI symptoms include the main manifestations, such as dizziness, headache, fatigue, blurred vision, chest tightness, palpitations, hand tremors, intolerance to movement, and even syncope especially in an upright position [49, 117] (IIa; B); (3) a positive HUTT or active standing test result indicating POTS is required (see section “Criteria for the positive response of active standing test and head-up tilt test”); and (4) other diseases that may cause similar symptoms should be excluded [65] (IIa; A).

#### Orthostatic hypotension

The diagnostic criteria of OH are as follows: (1) it is often associated with most of the above-mentioned inducing factors (as seen in VVS); (2) OI often occurs immediately after standing; (3) a positive HUTT or active standing test result is required (see Section “Criteria for the positive response of active standing test and head-up tilt test”); and (4) other diseases that cause similar symptoms should be excluded [68].

#### Orthostatic hypertension

The diagnostic criteria of OHT are as follows: (1) it is associated with the above predisposing factors in most patients (as seen in VVS). Being overweight, defined as having a body mass index (BMI) above the 85th percentile for the same sex and age group, causes a 6.07 times increase in the risk of OHT, while obesity (BMI > 95th percentile) increases the risk of OHT by 7.48 times. Drinking less water (water intake < 800 mL/day) causes a 4.03 times increase in the risk of OHT. Increased sleep duration by 1 hour per night decreases the risk of OHT by 74.3% [118] (IIa; B); (2) it is often associated with OI during the upright position; (3) a positive HUTT or active standing test result is required (see section “Criteria for the positive response of active standing test and head-up tilt test”); and (4) other diseases that cause similar symptoms should be excluded [66, 94, 119] (IIa; B).

#### Sitting tachycardia syndrome

The diagnostic criteria of STS are as follows: (1) it involves precipitating factors such as a sudden change in position (from supine to sitting), mental tension, stuffy environment, and insufficient sleep duration in most patients [48] (IIa; A); (2) the patients have symptoms of sitting intolerance, and syncope can also occur in severe cases; (3) the change of HR reaches the criteria for the positive response of active sitting test (see section “Criteria for the positive response of active sitting test”); and (4) other diseases that cause similar symptoms should be excluded [47, 48] (IIa; A).

#### Sitting hypertension

The diagnostic criteria of SHT are as follows: (1) it involves many precipitating factors such as a sudden change in position (from supine to sitting), mental tension, stuffy environment, and insufficient sleep time in most patients; (2) the patients have symptoms of sitting intolerance, and syncope can also occur in severe cases; (3) the change of BP reaches the criteria for the positive response of active sitting test (see section “Criteria for the positive response of active sitting test”); and (4) other diseases that cause similar symptoms should be excluded [47, 48] (IIa; A).

#### Situational syncope

SS is a type of reflex syncope, presupposed by special situations and directly related to relatively fixed triggers. Most SS episodes occur in the upright position and nearly half of the patients have positive responses to HUTT. SS includes micturition syncope, defecation syncope, cough syncope, swallowing syncope, and breath-holding spells according to different induced situations [57, 58] (IIa; B).

## Treatment

### Vasovagal syncope

Significant progress has been made in the individualized treatment of VVS [4, 120–125], and a common strategy to prevent the onset of VVS is non-pharmacological treatment based on lifestyle changes [28, 126] (IIa; B).

#### Health education

After VVS is diagnosed, health education on the management of syncope, including basic knowledge and skills in self-protection, is required for patients and legal guardians/parents, which can help reduce episodes of syncope and the possible physical as well as psychological harm it causes to pediatric patients. A health education model using mind mapping can significantly improve the effectiveness of this education [127] (IIa; B).

Trigger avoidance: children with VVS and their legal guardians/parents are advised to recognize common triggers, such as prolonged standing, quick position changes from a prolonged supine or sitting or squatting position to an upright position, sudden stops after prolonged movement (e.g., after long-distance running), a crowded or stuffy environment, and emotional stress (e.g., nervousness caused by painful stimuli or a medical operation). Syncope may be more likely to occur under some special conditions such as vomiting, diarrhea, anemia, low iron stores, infection, menstruation, and the use of some drugs, such as diuretics, which can reduce blood volume or BP. When triggers cannot be completely avoided, more attention should be given to pediatric patients to prevent physical injury and psychological disorders associated with syncope [10] (I; C).

Identification of presyncope symptoms and physical counter-pressure maneuvers: when presyncope occurs, patients should adjust their position, such as changing to a sitting position or lying down, if possible. Most symptoms can be alleviated in a short time [128] (IIa; B). Physical counter-pressure maneuvers may avoid or delay the syncope by increasing peripheral venous return, such as slight bending of the knees, contracting abdominal muscles or limb muscles (hands clasped, elbow flexion, legs crossed, and toes dorsiflexion) after prolonged standing [10, 129–131] (I; C).

Maintaining psychological well-being: recurrent syncope may adversely affect the psychological well-being of patients, seriously affecting their quality of life, and even causing PPS [42] (IIa; B). Therefore, legal guardians/parents and healthcare staff should pay attention to the mental health of the patients. Patients should be informed that this type of syncope usually has a favorable prognosis and should be approached with a positive mindset. Patients are encouraged to communicate with others to relieve psychological stress. Legal guardians/parents should be suggested to comfort and support their children. If necessary, special psychological counseling or therapy should be recommended [132, 133] (IIa; B).

Appropriate physical exercise: appropriate exercise is beneficial in increasing pump function of limb muscle in patients with VVS, which is important for recovery. Regular physical exercise plans and exercise without obvious discomfort are recommended. Additionally, children with VVS are advised to do physical exercise under supervision [131].

#### Autonomic nervous function exercise

##### Upright training (tilt training)

Patients are encouraged to stand upright against the wall with their feet approximately 15 cm away from the wall under supervision. The time for standing should be decided based on the tolerance and preference of the patient. Generally, the children may start from 5 minutes per session, twice daily, gradually increasing to 30 minutes per session [9] (I; C). Some biomarkers may help get a better effect of intervention from this measure [134–138] (IIa; B) (Table 4).

**Table 4:** Biomarkers for predicting therapeutic outcomes in children and adolescents with vasovagal syncope

Biomarkers and cutoff values	Clinical significance
ECG acceleration index < 26.77 [134]	Upright training (tilt training) may be recommended
Night-time DBP standard deviation < 5.75 mmHg [137]	Upright training (tilt training) may be recommended
Night-time DBP variation coefficient < 13.2% [137]	Upright training (tilt training) may be recommended
24-h urine adrenaline < 5.53 μg [138]	Upright training (tilt training) may be recommended
Body mass index < 18.9 kg/m^2^[144]	Oral rehydration salts may be recommended
24-h urine sodium < 83 mmoL [145]	Oral rehydration salts may be recommended
Flow-mediated vasodilation > 8.85% [150]	Midodrine hydrochloride may be recommended
Plasma CGRP level > 62.56 pg/mL [151]	Midodrine hydrochloride may be recommended
Serum uric acid < 299 μmol/L [152]	Midodrine hydrochloride may be recommended
HR increases before positive response in HUTT compared to the supine HR > 30 bpm [115]	Metoprolol may be recommended
24-h urinary norepinephrine > 34.84 μg [155]	Metoprolol may be recommended
Baroreflex sensitivity > 10 ms/mmHg [157]	Metoprolol may be recommended
LVEF > 70.5% [159]	Metoprolol may be recommended
LVFS > 38.5% [159]	Metoprolol may be recommended
Poincaré plot longitudinal/transverse axis ratio > 2.7 [160]	Metoprolol may be recommended

*ECG* electrocardiogram, *DBP* diastolic blood pressure, *CGRP* calcitonin gene-related peptide, *HR* heart rate, *HUTT* head-up tilt test, *LVEF* left ventricular ejection fraction, *LVFS* left ventricular fractional shortening

##### Dry towel wiping

The forearms and both legs of the patient are recommended to be repeatedly wiped with a soft and dry towel. Five minutes is recommended for each part, twice a day [139] (I; C).

#### Increase the intake of water and salt

Adequate daily water intake up to 30–50 mL/kg/day (no more than 1500–1700 mL) is recommended to achieve a clear urine color. Salt intake can be increased through additional salt intake in daily meals or regular drinking of oral rehydration salts (ORS) for at least two months, after which re-evaluation should be conducted for the patients [140–142] (IIa; B). In hot weather seasons, excessive sweating or fluid loss may occur and further increase of water and salt intake is needed. The combination of hemoglobin and BMI values can provide a reference for the selection of ORS [143] (IIa; B). Certain biomarkers can predict that ORS has a better effect on VVS [142, 144–146] (IIa; B) (Table 4). Additionally, ORS is especially suitable for children with VVS-VI [147] (IIa; B), but is not recommended for patients with hypertension, kidney disease, or heart failure [1] (I; C).

#### Pharmacological intervention

##### Indications

Children with recurrent syncope (more than twice every 6 months or more than three times per year), with risk of injury, and poor response to non-pharmacological therapy may be considered for pharmacological therapy [10, 148] (I; C).

##### Midodrine hydrochloride

The initial dose ranges from 1.25 to 2.5 mg/time, once or twice daily. After 2–4 weeks, it can be increased to 2.5 mg/time, three times daily. During medication, the supine BP should be monitored; the dose should be reduced or discontinued when the supine BP increases significantly (above the 95th percentile of BP) [149] (IIa; B). Some biomarkers can predict that midodrine hydrochloride may lead to better outcomes of VVS cases [150–152] (IIa; B) (Table 4). Children with a baseline BP above the 95th percentile of the same age and sex and children who are allergic to this medicine should not use midodrine hydrochloride as treatment.

##### Metoprolol

The initial dose is 0.5 mg/kg/day orally, twice daily, and can be gradually increased to a tolerable dose (no more than 2 mg/kg per day or the maximum adult daily dosage) [153] (IIa; A). Patients with significant sinus bradycardia, atrioventricular block (II or III degree), bronchial asthma, and allergy to drugs should be contraindicated to this therapy. Some biomarkers can predict the efficacy of metoprolol in such cases [115, 154–160] (IIa; B) (Table 4).

##### Other drugs

Fludrocortisone may reduce the probability of VVS syncope events in children and adolescents [161] (IIa; B). There is limited experience regarding the application of sertraline in children and adolescents with VVS [162] (IIa; B), and it can be considered when other drugs are ineffective; however, side effects must be carefully monitored.

#### Pacemaker therapy and others

Most children with VVS have a good prognosis [31] (I; C). For patients with recurrent syncope accompanied by prolonged cardiac arrest (> 4 seconds) and those surviving cardiopulmonary resuscitation [10, 163, 164], pacemaker implantation can be considered under the recommendation of pediatric cardiovascular specialists [11], as it may reduce the incidence of syncope events [165]. Left atrial ganglion catheter ablation can improve the symptoms of adults with VVS [166], and long-term follow-up of malignant VVS in children has achieved good results [167].

### Postural orthostatic tachycardia syndrome

The complicated pathophysiology and heterogeneous clinical manifestations of POTS pose a challenge to the treatment [168–172] (IIa; B) and require a comprehensive treatment plan [28]. The management of POTS in children and adolescents involves health education, pharmacological therapy, and other strategies [173–178]. Biomarker-oriented individualized therapy is an important strategy for the treatment of POTS [4, 124, 125, 179, 180] (IIa; B). However, it is also essential to focus on the management of the comorbidities [181] (IIa; B).

#### Health education

##### Avoidance of triggers

Patients should avoid prolonged standing, rapid changes in position from the supine or sitting or squatting to upright, taking drugs that aggravate symptoms, such as norepinephrine reuptake inhibitors, and being infected or fatigued [182] (IIa; B). Wearing compression garments can increase peripheral blood return and reduce orthostatic tachycardia caused by insufficient peripheral blood return [9] (I; C). At least 8 hours of sleep per day is also recommended [48, 173] (IIa; A). Patients with a salivary cortisol concentration > 4.1 ng/mL upon waking up in the morning had better sleep-stimulating treatment effects [183] (IIa; B).

##### Appropriate physical exercise

It is recommended to establish regular physical exercise plans for children and adolescents [184] (IIa; B) and ensure aerobic exercise for 1–2 hours/day for at least 5 days a week under parental supervision. Exercise can increase the blood volume of children, increase the left ventricular end-diastolic volume and stroke output, and enhance muscle pump function, thus improving the tolerance of children to upright positions. Some children with POTS have obvious exercise intolerance, and their exercise prescription should be gradual. Children who cannot tolerate long-term orthostatic exercise (such as long-distance running and ball sports) should start with non-orthostatic exercise. After 1–2 months of tolerating non-orthostatic exercise, they can gradually transition to orthostatic exercise and slowly prolong the time for exercise. A physical exercise plan should last for at least 3 months to form a lifestyle of exercise. However, other treatments should be considered for children with contraindications to sports [48] (IIa; A).

#### Autonomic nervous function exercise

The protocols of autonomic nervous function exercise are the same as the corresponding part of the treatment of VVS. Children and adolescents suffering from POTS with QTd > 43 ms are recommended to receive the autonomic nervous function exercise [185] (IIa; B).

#### Increase salt and water intake

For managing POTS in children and adolescents, salt and water intake should be increased [186] (IIa; B). Some biomarkers can predict the fact that salt and water intake have better effects on POTS [187–190] (IIa; B) (Table 5). For children and adolescents with severe symptoms, intravenous saline infusion may relieve symptoms [9] (I; C).

**Table 5:** Biomarkers for predicting therapeutic outcomes in children and adolescents with postural orthostatic tachycardia syndrome

Biomarkers and cutoff values	Clinical significance
MCHC > 347.5 g/L [179]	Increased intake of water and salt or oral rehydration salts may be recommended
24-h urinary sodium < 124 mmol [187]	Increased intake of water and salt or oral rehydration salts may be recommended
Body mass index < 18.02 kg/m^2^ [188]	Increased intake of water and salt or oral rehydration salts may be recommended
Increase in HR (from the supine position to standing) > 41 bpm [189]	Increased intake of water and salt or oral rehydration salts may be recommended
The maximum HR during active standing test > 123 bpm [189]	Increased intake of water and salt or oral rehydration salts may be recommended
Baroreflex sensitivity > 17.01 ms/mmHg [190]	Increased intake of water and salt or oral rehydration salts may be recommended
Plasma copeptin level > 10.5 pmol/L [192]	Midodrine hydrochloride may be recommended
Increase in SBP (from the supine position to upright) ≤ 0 mmHg [193]	Midodrine hydrochloride may be recommended
Increase in DBP (from the supine position to upright) ≤ 6.5 mmHg [193]	Midodrine hydrochloride may be recommended
Flow-mediated vasodilation > 9.85% [196]	Midodrine hydrochloride may be recommended
Erythrocyte hydrogen sulfide production > 27.1 nmol/min/10^8^ RBC [197]	Midodrine hydrochloride may be recommended
Plasma MR-proADM level > 61.5 pg/mL [199]	Midodrine hydrochloride may be recommended
Poincaré plot longitudinal axis < 573.9 ms and transverse/longitudinal axis ratio > 2.9 [200]	Midodrine hydrochloride may be recommended
Plasma copeptin level < 10.2 pmol/L [203]	Metoprolol may be recommended
Plasma C-type natriuretic peptide > 32.55 pg/mL [204]	Metoprolol may be recommended
Orthostatic plasma norepinephrine level > 3.59 pg/mL [205]	Metoprolol may be recommended
Baroreflex sensitivity > 8.045 ms/mmHg [206]	Metoprolol may be recommended
HR variability TR ≤ 33.7 and SDNN index ≤ 79.0 ms [207]	Metoprolol may be recommended
Baseline QT interval dispersion > 37 ms [208]	Metoprolol may be recommended
Corrected QT interval dispersion > 47.9 ms [208]	Metoprolol may be recommended
Corrected maximum P-wave duration > 109 ms, corrected minimum QT interval > 382.5 ms, and T-wave apex to T-wave end interval dispersion > 45.6 ms [209]	Metoprolol may be recommended
5-min HR at HUTT > 110 bpm [210]	Metoprolol may be recommended
10-min HR at HUTT> 112 bpm [210]	Metoprolol may be recommended
5-min HR difference from baseline HR at HUTT > 34 bpm [210]	Metoprolol may be recommended
10-min HR difference from baseline HR at HUTT > 37 bpm [210]	Metoprolol may be recommended
The product of HR and BP at 5-min during HUTT > 11,548.5 bpm × mmHg [211]	Metoprolol may be recommended
The product of HR and BP at 10-min during HUTT > 10,988.0 bpm × mmHg [211]	Metoprolol may be recommended

*MCHC* mean erythrocyte hemoglobin concentration, *HR* heart rate, *BP* blood pressure, *SBP* systolic blood pressure, *DBP* diastolic blood pressure, *RBC* red blood cell, *MR-proADM* mid-regional fragment of pro-adrenomedullin, *TR* triangulation index, *SDNN* standard deviation of all NN intervals, *HUTT* head-up tilt test

#### Pharmacological intervention

Pharmacological intervention should be considered for children and adolescents with severe symptoms that significantly affect the quality of life.

##### Midodrine hydrochloride

The initial dose ranges from 1.25 to 2.5 mg/time orally, once or twice per day, and can be switched to 2.5 mg/time, three times per day after 2–4 weeks if the effect is not observed. Also, special attention should be paid to BP monitoring [191–197] (IIa; B). Some biomarkers can predict the effects of midodrine hydrochloride prior to treating POTS [191–200] (IIa; B) (Table 5). Generally, midodrine hydrochloride may be more effective than metoprolol or ORS in the treatment of children and adolescents with POTS [201] (IIa; B). The best curative effect can be achieved after 3 months of treatment, while extending the treatment course to 6 months does not significantly improve the therapeutic effect [202] (IIa; B). The contraindications are mentioned above (see section “Pharmacological intervention” of “Vasovagal syncope”).

##### Metoprolol

The initial dose is 0.5 mg/kg/day orally, twice per day. This dosage can be gradually increased to a tolerable dose after 2–4 weeks but should be no more than 2mg/kg per day or the maximum adult daily dosage [1]. Some biomarkers can predict the effects of metoprolol [203–212] (IIa; B) (Table 5). A model with corrected maximum P-wave duration (ms), corrected minimum QT interval (ms), and Tped (ms) in ECG can predict the efficacy of metoprolol for children with POTS [209] (IIa; B). Metoprolol can reduce clinical symptom scores [201] (IIa; B). The contraindications are mentioned above (see section “Pharmacological intervention” of “Vasovagal syncope”).

#### Management of comorbidities

Correcting iron and vitamin deficiencies may be beneficial in improving the symptoms of patients with POTS [213–215] (IIa; B). The evaluation and management of POTS comorbidities are also receiving increasing attention [182, 216]. Other common comorbidities of POTS include allergic diseases, migraines, mental disorders, sleeping disorders, hyperventilation syndrome, chronic fatigue syndrome, joint hypermobility syndrome, and gastrointestinal disorders. The treatment of POTS in children requires a comprehensive assessment of the comorbidities [184] (IIa; B).

### Orthostatic hypotension

OH can be managed firstly using non-pharmacological measures, such as avoiding triggers, increasing water and salt intake, taking physical counter-pressure maneuvers, and wearing elastic socks [217–219] (IIa; B). If non-pharmacological treatments are not effective, midodrine hydrochloride or fludrocortisone can be considered [2] (I; C).

### Orthostatic hypertension

Non-pharmacological therapy [220], including health education and autonomic function exercise, can be used. Health education includes knowledge on avoiding prolonged standing or sudden changes in position (from supine or sitting or squatting to an upright position) and providing psychological assistance. Currently, no medicines have been recommended for the treatment of OHT in children. However, special attention should be paid to the possibility of developing primary hypertension during late adolescence or adulthood in children with OHT.

### Other types of neurally mediated syncope

Children and adolescents with SS usually experience recurrent syncope under specific circumstances [57, 58] (IIa; B). For example, the treatment of micturition syncope is health education. It is suggested that the patient should not stand up abruptly when getting up in the morning and avoid holding urine for a long period. It is recommended to micturate in a squatting position. If a syncopal attack occurs during micturition, the witness should assist the patient to lay down in a safe and well-ventilated environment and retain a unobstructed airway for the patient. For children with breath-holding spells, only health education and an explanation of the prognosis of the disease are needed, which means that the parents should understand that the attacks are generally not life-threatening. During a breath-holding spell, the parents need to ensure that their child lies on his or her side to prevent trauma and aspiration.

### Follow-up

The prognosis of most children and adolescents with VVS or POTS is generally good, but regular follow-up is still needed to get better outcomes [221–223] (IIa; B). A follow-up at 1–3 months after the initial diagnosis and treatment is recommended, and the interval for follow-up thereafter should depend on the symptoms of the patients. Children and adolescents with more than four episodes of syncope have a reduced symptom-free rate [224] (IIa; B), while those with cardiac arrest or convulsive events during HUTT do not indicate a poor prognosis [81] (IIa; B).

In the follow-up of VVS, the frequency and manifestations of symptoms, treatment compliance, and drug tolerance should be recorded. The recurrent onset of symptoms is the main factor affecting the quality of life of children and adolescents [132] (IIa; B). Supine mean arterial pressure in HUTT, baseline urine specific gravity, the standard deviation of average RR intervals in milliseconds, mean corpuscular hemoglobin, and demographic factors are associated with the risk of syncope or presyncope recurrence [80, 225, 226] (IIa; B). The evaluation of therapeutic efficacy should mainly focus on symptom control [205] (IIa; B). A symptom score, which is based on the frequency of syncopal episodes, has been used to evaluate the efficacy [143] (IIa; B). A negative response to HUTT is not recommended as an effective indicator for treatment.

In the follow-up of children with POTS, the OI symptom score is recommended as an indicator to judge the therapeutic effect, and a score reduction of ≥ 2 points is considered effective [192] (IIa; B). If the treatment effect is not good, the condition of the patient should be re-evaluated to ensure that the diagnosis is correct and the treatment plan adjusted [227] (IIa; B). In addition, the influence of comorbidity should be considered. The duration of symptoms before treatment and the maximum upright HR in a standing position can be used as independent indicators to estimate the long-term prognosis, with patients receiving conventional intervention for POTS generally experiencing satisfactory long-term outcomes [228] (IIa; B). A baseline plasma mid-regional fragment of pro-adrenomedullin > 61.5 ng/L indicates that midodrine hydrochloride has a favorable long-term effect [229] (IIa; B). In a questionnaire survey, a total of 86% of children and adolescents with POTS showed symptomatic remission 5 years after their initial diagnosis [230] (IIa; B).

## Conclusions

In summary, based on the latest global research reports, the guidelines were updated. In terms of the diagnosis of NMS as well as its related disorders in children, the concepts of STS and SHT were introduced; and the comorbidities of NMS were emphasized. Regarding the management, the detailed rules and recommendations for some non-pharmacological therapies were refined and the management of comorbidities was highlighted. Specially, the newly discovered biomarkers that can be used to guide individualized treatment in recent years were summarized and recommended. Furthermore, specific suggestions were put forward for the follow-up process and efficacy evaluation. The new guidelines will provide comprehensive guidance and reference for the diagnosis and treatment of NMS in children and adolescents. It is expected that the guidelines will be further improved and perfected in the future clinical practice and related studies.

## Data Availability

The data are available from the corresponding author on reasonable request.

## References

[1] Wang C, Li Y, Liao Y, Tian H, Huang M, Dong X, et al. 2018 Chinese Pediatric Cardiology Society (CPCS) guideline for diagnosis and treatment of syncope in children and adolescents. Sci Bull (Beijing). 2018;63:1558–64. 36751076 10.1016/j.scib.2018.09.019

[2] Brignole M, Moya A, de Lange FJ, Deharo JC, Elliott PM, Fanciulli A, et al. 2018 ESC guidelines for the diagnosis and management of syncope. Eur Heart J. 2018;39:1883–948. 29562304 10.1093/eurheartj/ehy037

[3] Kenny RA, Brignole M, Dan GA, Deharo JC, van Dijk JG, Doherty C, et al. Syncope unit: rationale and requirement—the European Heart Rhythm Association position statement endorsed by the Heart Rhythm Society. Europace. 2015;17:1325–40. 26108809 10.1093/europace/euv115

[4] Tao C, Tang C, Jin H, Du J. Pediatric syncope: a hot issue in focus. Sci Bull (Beijing). 2020;65:513–5. 36659179 10.1016/j.scib.2019.12.026

[5] Hu E, Liu X, Chen Q, Wang C. Investigation on the incidence of syncope in children and adolescents aged 2–18 years in Changsha. Front Pediatr. 2021;9:638394. 33829003 10.3389/fped.2021.638394 PMC8019745

[6] Ruwald MH, Hansen ML, Lamberts M, Hansen CM, Højgaard MV, Køber L, et al. The relation between age, sex, comorbidity, and pharmacotherapy and the risk of syncope: a Danish nationwide study. Europace. 2012;14:1506–14. 22588456 10.1093/europace/eus154

[7] Chen L, Li X, Todd O, Wang C, Jin H, Du J. A clinical manifestation-based prediction of haemodynamic patterns of orthostatic intolerance in children: a multi-centre study. Cardiol Young. 2014;24:649–53. 23866994 10.1017/S1047951113000929

[8] Kara A, Doğan MT. The psychopathology, depression, and anxiety levels of children and adolescents with vasovagal syncope: a case-control study. J Nerv Ment Dis. 2021;209:547–51. 33840767 10.1097/NMD.0000000000001334

[9] Sheldon RS, Grubb BP 2nd, Olshansky B, Shen WK, Calkins H, Brignole M, et al. 2015 Heart Rhythm Society expert consensus statement on the diagnosis and treatment of postural tachycardia syndrome, inappropriate sinus tachycardia, and vasovagal syncope. Heart Rhythm. 2015;12:e41–63. 25980576 10.1016/j.hrthm.2015.03.029 PMC5267948

[10] Shen WK, Sheldon RS, Benditt DG, Cohen MI, Forman DE, Goldberger ZD, et al. 2017 ACC/AHA/HRS guideline for the evaluation and management of patients with syncope: a report of the american college of cardiology/american heart association task force on clinical practice guidelines, and the heart rhythm society. J Am Coll Cardiol. 2017;70:e39–110. 28286221 10.1016/j.jacc.2017.03.003

[11] Sanatani S, Chau V, Fournier A, Dixon A, Blondin R, Sheldon RS. Canadian Cardiovascular Society and Canadian Pediatric Cardiology Association Position Statement on the approach to syncope in the pediatric patient. Can J Cardiol. 2017;33:189–98. 27838109 10.1016/j.cjca.2016.09.006

[12] Wang Y, Wang Y, He B, Tao C, Han Z, Liu P, et al. Plasma human growth cytokines in children with vasovagal syncope. Front Cardiovasc Med. 2022;9:1030618. 36312268 10.3389/fcvm.2022.1030618 PMC9614254

[13] Zhang Q, Sun Y, Zhang C, Qi J, Du J. Vitamin D deficiency and vasovagal syncope in children and adolescents. Front Pediatr. 2021;9:575923. 33732666 10.3389/fped.2021.575923 PMC7959715

[14] Zhang Q, Li Y, Liao Y, Du J. Significance of red cell distribution width in the differential diagnosis between neurally mediated syncope and arrhythmic syncope in children. Cardiol Young. 2017;27:691–6. 27434230 10.1017/S1047951116001098

[15] Bai W, Chen S, Tang CS, Qi JG, Cui QH, Xu M, et al. Gut microbiota analysis and its significance in vasovagal syncope in children. Chin Med J (Engl). 2019;132:411–9. 30707176 10.1097/CM9.0000000000000086 PMC6595724

[16] Chen L, Zhang CY, Du JB. Diagnostic values of heart rate variability on unexplained syncope in children. Beijing Da Xue Xue Bao Yi Xue Ban. 2013;45:761–5 (**in Chinese**). 24136274

[17] Cui YX, Du JB, Jin HF. Insights into postural orthostatic tachycardia syndrome after COVID-19 in pediatric patients. World J Pediatr. 2024;20:201–7. 38363488 10.1007/s12519-024-00796-0

[18] Jin HF, Yang JY, Li XY, Zhu LL, Han L, Zhang FW, et al. A modified Calgary syncope syndrome score in the differential diagnosis between cardiac syncope and vasovagal syncope. Zhonghua Er Ke Za Zhi. 2012;50:117–20 (**in Chinese**). 22455635

[19] Yang J, Zhu L, Chen S, Li X, Zhang Q, Zhang F, et al. Modified Calgary score in differential diagnosis between cardiac syncope and postural orthostatic tachycardia syndrome-associated syncope in children. Cardiol Young. 2013;23:400–4. 23046517 10.1017/S1047951112001266

[20] Zou R, Wang S, Zhu L, Wu L, Lin P, Li F, et al. Calgary score and modified Calgary score in the differential diagnosis between neurally mediated syncope and epilepsy in children. Neurol Sci. 2017;38:143–9. 27747448 10.1007/s10072-016-2740-5

[21] Li Y, Liu J, Wang M, Zhao H, Liu X, Hu J, et al. Predictive value of EGSYS score in the differential diagnosis of cardiac syncope and neurally mediated syncope in children. Front Cardiovasc Med. 2023;10:1091778. 37008325 10.3389/fcvm.2023.1091778 PMC10063910

[22] Zhang J, Tang HN, Wang YW, Li F, Cai H, Lin P, et al. Predictive value of blood cell parameters in the diagnosis of vasovagal syncope in children. Zhonghua Er Ke Za Zhi. 2022;60:792–7 (**in Chinese**). 35922190 10.3760/cma.j.cn112140-20220129-00098

[23] Zhao J, Du JB. Blood pressure variability in children with autonomous nerve mediated syncope. Zhonghua Er Ke Za Zhi. 2012;50:712–3 (**in Chinese**). 23158826

[24] Zou R, Wang S, Cai H, Li F, Lin P, Wang Y, et al. Vitamin D deficiency in children with vasovagal syncope is associated with impaired circadian rhythm of blood pressure. Front Neurosci. 2021;15:712462. 34456677 10.3389/fnins.2021.712462 PMC8387869

[25] Li J, Zhang Q, Gao J, Jin H, Du J. Significance of serum iron in the differential diagnosis between vasovagal syncope and postural orthostatic tachycardia syndrome in children. Beijing Da Xue Xue Bao Yi Xue Ban. 2013;45:923–7 (**in Chinese**). 24343075

[26] Jin HF, Du JB. Development and future direction of clinical diagnosis and management of pediatric syncope. Zhonghua Er Ke Za Zhi. 2020;58:88–90 (**in Chinese**). 32102142 10.3760/cma.j.issn.0578-1310.2020.02.003

[27] Chen L, Wang C, Wang H, Tian H, Tang C, Jin H, et al. Underlying diseases in syncope of children in China. Med Sci Monit. 2011;17:PH49–53. 10.12659/MSM.881795 PMC3539540 21629199

[28] Cui Y, Liao Y, Zhang Q, Yan H, Liu P, Wang Y, et al. Spectrum of underlying diseases in syncope and treatment of neurally-mediated syncope in children and adolescents over the past 30 years: a single center study. Front Cardiovasc Med. 2022;9:1017505. 36518687 10.3389/fcvm.2022.1017505 PMC9742595

[29] Li HX, Gao L, Yuan Y. Advance in the understanding of vasovagal syncope in children and adolescents. World J Pediatr. 2021;17:58–62. 32405708 10.1007/s12519-020-00367-z

[30] Zhang QY, Karmane SI, Du JB. Physiologic neurocirculatory patterns in the head-up tilt test in children with orthostatic intolerance. Pediatr Int. 2008;50:195–8. 18353058 10.1111/j.1442-200X.2008.02556.x

[31] Moya A, Sutton R, Ammirati F, Blanc JJ, Brignole M, Dahm JB, et al. Guidelines for the diagnosis and management of syncope (version 2009). Eur Heart J. 2009;30:2631–71. 19713422 10.1093/eurheartj/ehp298 PMC3295536

[32] Zhang W, Wang C, Zou R, Liu L, Wu L, Luo X, et al. Changes in P-wave, T-wave, and ST segment amplitude in 12 lead electrocardiogram in children with breath holding spell. Zhong Nan Da Xue Xue Bao Yi Xue Ban. 2016;41:600–5 (**in Chinese**). 27374444 10.11817/j.issn.1672-7347.2016.06.008

[33] Wang YR, Li XY, Du JB, Sun Y, Xu WR, Wang YL, et al. Impact of comorbidities on the prognosis of pediatric vasovagal syncope. World J Pediatr. 2022;18:624–8. 35608720 10.1007/s12519-022-00566-w

[34] Liao Y, Zhang QY, Li HX, Wang YL, Liu P, Du JB. Co-morbidity of vasovagal syncope and postural tachycardia syndrome with allergic diseases in children. Beijing Da Xue Xue Bao Yi Xue Ban. 2017;49:783–8 (**in Chinese**). 29045956

[35] Wang Y, Du J, Jin H, Liao Y. Comorbidity of neurally mediated syncope and allergic disease in children. Front Immunol. 2020;11:1865. 32983103 10.3389/fimmu.2020.01865 PMC7485378

[36] Quan W, Wang Y, Chen S, Du J. Orthostatic intolerance and coagulation abnormalities: an update. Neurosci Bull. 2019;35:171–7. 30315398 10.1007/s12264-018-0295-6 PMC6357271

[37] Zou R, Wang S, Liu P, Chen D, Yan J, Cai H, et al. The association between patent foramen ovale and unexplained syncope in pediatric patients. Ital J Pediatr. 2024;50:2. 38185629 10.1186/s13052-023-01572-y PMC10773133

[38] Liao Y, Qi JG, Yan H, Zhang QY, Ji TY, Chang XZ, et al. Comorbidity of chronic fatigue syndrome, postural tachycardia syndrome, and narcolepsy with 5,10-methylenetetrahydrofolate reductase (*MTHFR*) mutation in an adolescent: a case report. Chin Med J (Engl). 2021;134:1495–7. 33788782 10.1097/CM9.0000000000001387 PMC8213257

[39] Gao Y, Zhang Q, Sun Y, Du J. Congenital anomalous origin of coronary artery disease in children with syncope: a case series. Front Pediatr. 2022;10:879753. 35865709 10.3389/fped.2022.879753 PMC9294363

[40] Chen L, Zhang Q, Ingrid S, Chen J, Qin J, Du J. Aetiologic and clinical characteristics of syncope in Chinese children. Acta Paediatr. 2007;96:1505–10. 17714543 10.1111/j.1651-2227.2007.00446.x

[41] Liao Y, Du J, Benditt DG, Jin H. Vasovagal syncope or psychogenic pseudosyncope: a major issue in the differential diagnosis of apparent transient loss of consciousness in children. Sci Bull (Beijing). 2022;67:1618–20. 36546036 10.1016/j.scib.2022.07.024

[42] Zhang Z, Jiang X, Han L, Chen S, Tao L, Tao C, et al. Differential diagnostic models between vasovagal syncope and psychogenic pseudosyncope in children. Front Neurol. 2020;10:1392. 32038462 10.3389/fneur.2019.01392 PMC6989585

[43] Li C, Zhang Y, Liao Y, Han L, Zhang Q, Fu J, et al. Differential diagnosis between psychogenic pseudosyncope and vasovagal syncope in children: a quantitative scoring model based on clinical manifestations. Front Cardiovasc Med. 2022;9:839183. 35155640 10.3389/fcvm.2022.839183 PMC8829042

[44] Li YW, Chen L, Du JB, Yang YY, Jin HF. Cost-effectiveness of diagnostic approaches to vasovagal syncope. Chin Med J (Engl). 2010;123:2635–9. 21034644

[45] Chen L, Yang YY, Wang C, Wang HW, Tian H, Zhang QY, et al. A multi-center study of hemodynamic characteristics exhibited by children with unexplained syncope. Chin Med J (Engl). 2006;119:2062–8. 17199957

[46] Tretter JT, Kavey RE. Distinguishing cardiac syncope from vasovagal syncope in a referral population. J Pediatr. 2013;163:1618–23. 23992679 10.1016/j.jpeds.2013.07.023

[47] Tao C, Han Z, Yan Y, Pan Z, Zhu H, Li X, et al. Sitting-induced hemodynamic changes and association with sitting intolerance in children and adolescents: a cross-sectional study. Sci Rep. 2020;10:13921. 32811875 10.1038/s41598-020-70925-y PMC7435175

[48] Wang Y, Han Z, Wang Y, Yan Y, Pan Z, Zhu H, et al. Risk factors of sitting-induced tachycardia syndrome in children and adolescents. PLoS One. 2022;17:e0265364. 35303039 10.1371/journal.pone.0265364 PMC8932569

[49] Wang YY, Du JB, Jin HF. Diferential diagnosis of vasovagal syncope and postural tachycardia syndrome in children. World J Pediatr. 2020;16:549–52. 32020440 10.1007/s12519-019-00333-4

[50] Zhang Q, Jin H, Qi J, Yan H, Du J. Diagnostic value of serum brain natriuretic peptide in syncope in children and adolescents. Acta Paediatr. 2013;102:e210–4. 23373852 10.1111/apa.12182

[51] Wang X, Wang S, Xiao H, Zou R, Cai H, Liu L, et al. The value of QT interval in differentiating vasovagal syncope from epilepsy in children. Ital J Pediatr. 2022;48:197. 36510267 10.1186/s13052-022-01388-2 PMC9743691

[52] Lin J, Zhao H, Ma L, Jiao F. Body mass index is decreased in children and adolescents with postural tachycardia syndrome. Turk J Pediatr. 2019;61:52–8. 31559722 10.24953/turkjped.2019.01.009

[53] Wang Y, Zhang C, Chen S, Li X, Jin H, Du J. Frequency domain indices of heart rate variability are useful for differentiating vasovagal syncope and postural tachycardia syndrome in children. J Pediatr. 2019;207:59–63. 30626483 10.1016/j.jpeds.2018.11.054

[54] Tao C, Chen S, Li H, Wang Y, Wang Y, Liu P, et al. Value of immediate heart rate alteration from supine to upright in differential diagnosis between vasovagal syncope and postural tachycardia syndrome in children. Front Pediatr. 2018;6:343. 30510926 10.3389/fped.2018.00343 PMC6252323

[55] Zhang F, Li X, Stella C, Chen L, Liao Y, Tang C, et al. Plasma hydrogen sulfide in differential diagnosis between vasovagal syncope and postural orthostatic tachycardia syndrome in children. J Pediatr. 2012;160:227–31. 21920536 10.1016/j.jpeds.2011.08.008

[56] Yuan P, Lian Z, Wang Y, Wang Y, Zhang C, Du J, et al. Poincaré plot is useful for distinguishing vasovagal syncope from postural tachycardia syndrome in children. Front Pediatr. 2022;10:758100. 35372154 10.3389/fped.2022.758100 PMC8965582

[57] Zou R, Wang S, Lin P, Hu C, Wang Y, Li F, et al. The clinical characteristics of situational syncope in children and adults undergoing head-up tilt testing. Am J Emerg Med. 2020;38:1419–23. 31843331 10.1016/j.ajem.2019.11.042

[58] Trivedi MK, Arora G. Micturition syncope with asystole in a paediatric patient. Cardiol Young. 2021;31:661–2. 33308342 10.1017/S1047951120004473

[59] Choi YJ, Kang KW, Jang SH, Kim JG, Lee SJ, Jung KT. Heart rate recovery and diastolic blood pressure ratio on the treadmill test predict an induction and recurrence of vasovagal syncope. Korean J Intern Med. 2019;34:315–23. 29240992 10.3904/kjim.2017.180 PMC6406081

[60] Zhang Q, Zhu L, Wang C, Du Z, Hu X, Tian H, et al. Value of history taking in children and adolescents with cardiac syncope. Cardiol Young. 2013;23:54–60. 22417947 10.1017/S1047951112000303

[61] Stewart JM. Common syndromes of orthostatic intolerance. Pediatrics. 2013;131:968–80. 23569093 10.1542/peds.2012-2610 PMC3639459

[62] Placidi S, Drago F, Milioni M, Verticelli L, Tamburri I, Silvetti MS, et al. Miniaturized implantable loop recorder in small patients: an effective approach to the evaluation of subjects at risk of sudden death. Pacing Clin Electrophysiol. 2016;39:669–74. 27062386 10.1111/pace.12866

[63] Huang YJ, Zhou ZW, Xu M, Ma QW, Yan JB, Wang JY, et al. Alteration of gene expression profiling including *GPR174* and *GNG2* is associated with vasovagal syncope. Pediatr Cardiol. 2015;36:475–80. 25367286 10.1007/s00246-014-1036-x

[64] Zhang QY, Du JB, Chen JJ, Li WZ. Association of clinical characteristics of unexplained syncope with the outcome of head-up tilt tests in children. Pediatr Cardiol. 2004;25:360–4. 14727100 10.1007/s00246-003-0513-4

[65] Li H, Han Z, Chen S, Liao Y, Wang Y, Liu P, et al. Total peripheral vascular resistance, cardiac output, and plasma C-type natriuretic peptide level in children with postural tachycardia syndrome. J Pediatr. 2015;166:1385–9.e1–2. 10.1016/j.jpeds.2015.03.032 25890678

[66] Zhao J, Han Z, Zhang X, Du S, Liu AD, Holmberg L, et al. A cross-sectional study on upright heart rate and BP changing characteristics: basic data for establishing diagnosis of postural orthostatic tachycardia syndrome and orthostatic hypertension. BMJ Open. 2015;5:e007356. 26033944 10.1136/bmjopen-2014-007356 PMC4458681

[67] Cai H, Wang S, Zou R, Li F, Zhang J, Wang Y, et al. Diagnostic value of diurnal variability of orthostatic heart rate increment in children and adolescents with POTS. Front Pediatr. 2021;9:644461. 34055686 10.3389/fped.2021.644461 PMC8157922

[68] Ricci F, De Caterina R, Fedorowski A. Orthostatic hypotension: epidemiology, prognosis, and treatment. J Am Coll Cardiol. 2015;66:848–60. 26271068 10.1016/j.jacc.2015.06.1084

[69] Zhang Q, Li J, Xie Y, Zhao J, Du J. Orthostatic hypertension in children and adolescents with postural tachycardia syndrome. J Trap Pediatr. 2014;60:461–6. 10.1093/tropej/fmu055 25326283

[70] Wang Y, Xu Y, Li F, Lin P, Zhang J, Zou R, et al. Diagnostic and prognostic value of T-wave amplitude difference between supine and orthostatic electrocardiogram in children and adolescents with postural orthostatic tachycardia syndrome. Ann Noninvasive Electrocardiol. 2020;25:e12747. 32112609 10.1111/anec.12747 PMC7358833

[71] Tan C, Yi X, Chen Y, Wang S, Ji Q, Li F, et al. The changes of T-wave amplitude and QT interval between the supine and orthostatic electrocardiogram in children with dilated cardiomyopathy. Front Pediatr. 2021;9:680923. 34295860 10.3389/fped.2021.680923 PMC8290918

[72] Macedo PG, Leite LR, Santos-Neto L, Hachul D. Tilt test—from the necessary to the indispensable. Arq Bras Cardiol. 2011;96:246–54 (**in English, Portuguese, Spanish**). 21271173 10.1590/s0066-782x2011005000006

[73] Robinson JA, Shivapour JK, Snyder CS. Tilt table testing to diagnose pseudosyncope in the pediatric population. Congenit Heart Dis. 2017;12:411–6. 28240408 10.1111/chd.12458

[74] Lin J, Wang Y, Ochs T, Tang C, Du J, Jin H. Tilt angles and positive response of head-up tilt test in children with orthostatic intolerance. Cardiol Young. 2015;25:76–80. 24124665 10.1017/S1047951113001601

[75] Thijs RD, Brignole M, Falup-Pecurariu C, Fanciulli A, Freeman R, Guaraldi P, et al. Recommendations for tilt table testing and other provocative cardiovascular autonomic tests in conditions that may cause transient loss of consciousness: consensus statement of the European Federation of Autonomic Societies (EFAS) endorsed by the American Autonomic Society (AAS) and the European Academy of Neurology (EAN). Clin Auton Res. 2021;31:369–84. 33740206 10.1007/s10286-020-00738-6 PMC8184725

[76] Zou R, Wang S, Li F, Lin P, Zhang J, Wang Y, et al. The application of head-up tilt test to diagnose hemodynamic type of orthostatic intolerance in children aged between 3 to 5 years old. Front Pediatr. 2021;9:623880. 33748043 10.3389/fped.2021.623880 PMC7965941

[77] Li W, Wang C, Wu LJ, Hu CY, Xu Y, Li MX, et al. Arrhythmia after a positive head-up tilt table test. Zhonghua Xin Xue Guan Bing Za Zhi. 2010;38:805–8 (**in Chinese**). 21092649

[78] Chu W, Wang C, Lin P, Li F, Wu L, Xie Z. Transient aphasia: a rare complication of head-up tilt test. Neurol Sci. 2014;35:1127–32. 24514919 10.1007/s10072-014-1664-1

[79] Wang C, Li W, Wu L, Lin P, Li F, Luo H, et al. Clinical characteristics and treatment of 89 patients with head-up tilt table test induced syncope with convulsion. Zhong Nan Da Xue Xue Bao Yi Xue Ban. 2013;38:70–3 (**in Chinese**). 23406867 10.3969/j.issn.1672-7347.2013.01.013

[80] Wang S, Peng Y, Zou R, Wang Y, Cai H, Li F, et al. The relationship between demographic factors and syncopal symptom in pediatric vasovagal syncope. Sci Rep. 2023;13:22724. 38123593 10.1038/s41598-023-49722-w PMC10733366

[81] Zou R, Wang S, Wen W, Cai H, Wang Y, Liu P, et al. Risk factors and prognostic follow-up of vasovagal syncope children with seizure-like activities during head-up tilt test induced-syncope. Front Cardiovasc Med. 2022;9:916542. 35757321 10.3389/fcvm.2022.916542 PMC9226399

[82] Liao D, Xu Y, Zou R, Wu L, Luo X, Li F, et al. The circadian rhythm of syncopal episodes in patients with neurally mediated syncope. Int J Cardiol. 2016;215:186–92. 27128529 10.1016/j.ijcard.2016.04.086

[83] Li H, Liao Y, Han Z, Wang Y, Liu P, Zhang C, et al. Head-up tilt test provokes dynamic alterations in total peripheral resistance and cardiac output in children with vasovagal syncope. Acta Paediatr. 2018;107:1786–91. 29603793 10.1111/apa.14342

[84] Liao Y, Chen S, Liu X, Zhang Q, Ai Y, Wang Y, et al. Flow-mediated vasodilation and endothelium function in children with postural orthostatic tachycardia syndrome. Am J Cardiol. 2010;106:378–82. 20643249 10.1016/j.amjcard.2010.03.034

[85] Zhang QY, Du JB, Li WZ. Head-up tilt testing potentiated with sublingual nitroglycerin for the diagnosis of unexplained syncope in children. Zhonghua Er Ke Za Zhi. 2004;42:371–4 (**in Chinese**). 15189698

[86] Zhang W, Zou R, Wu L, Luo X, Xu Y, Li F, et al. The changes of electrolytes in serum and urine in children with neurally mediated syncope cured by oral rehydration salts. Int J Cardiol. 2017;233:125–9. 28043672 10.1016/j.ijcard.2016.12.138

[87] Zhang Q, Du J, Wang C, Du Z, Wang L, Tang C. The diagnostic protocol in children and adolescents with syncope: a multi-centre prospective study. Acta Paediatr. 2009;98:879–84. 19183119 10.1111/j.1651-2227.2008.01195.x

[88] Liao Y, Xu WR, Li HX, Tang CS, Jin HF, Du JB. Plasma neuropeptide Y levels in vasovagal syncope in children. Chin Med J (Engl). 2017;130:2778–84. 29176136 10.4103/0366-6999.219157 PMC5717855

[89] Wang S, Peng Y, Zou R, Liao D, Yan J, Chen D, et al. Relationship between hemodynamic type and syncopal symptoms in pediatric vasovagal syncope. Eur J Pediatr. 2024;183:179–84. 37855929 10.1007/s00431-023-05278-5

[90] Xu WR, Jin HF, Du JB. Diagnosis and treatment of malignant vasovagal syncope in children. Zhonghua Er Ke Za Zhi. 2022;60:64–6 (**in Chinese**). 34986627 10.3760/cma.j.cn112140-20211018-00883

[91] Sun R, Kang YY, Zhang MM, Li AJ, Lin Y, Shi L, et al. Risk factors associated with malignant vasovagal syncope in children. Zhonghua Er Ke Za Zhi. 2023;61:131–5 (**in Chinese**). 36720594 10.3760/cma.j.cn112140-20221217-01052

[92] Brewster JA, Garland EM, Biaggioni I, Black BK, Ling JF, Shibao CA, et al. Diurnal variability in orthostatic tachycardia: implications for the postural tachycardia syndrome. Clin Sci (Lond). 2012;122:25–31. 21751966 10.1042/CS20110077 PMC3172399

[93] Moon J, Lee HS, Byun JI, Sunwoo JS, Shin JW, Lim JA, et al. The complexity of diagnosing postural orthostatic tachycardia syndrome: influence of the diurnal variability. J Am Soc Hypertens. 2016;10:263–70. 26857333 10.1016/j.jash.2016.01.011

[94] Hu Y, Wang Y, He B, Wang Y, Han Z, Tao C, et al. Sympathetic overactivation from supine to upright is associated with orthostatic hypertension in children and adolescents. Front Pediatr. 2020;8:54. 32154199 10.3389/fped.2020.00054 PMC7047410

[95] Zhao J, Yang J, Du S, Tang C, Du J, Jin H. Changes of atrial natriuretic peptide and antidiuretic hormone in children with postural tachycardia syndrome and orthostatic hypertension: a case control study. Chin Med J (Engl). 2014;127:1853–7. 24824244

[96] Lee H, Low PA, Kim HA. Patients with orthostatic intolerance: relationship to autonomic function tests results and reproducibility of symptoms on tilt. Sci Rep. 2017;7:5706. 28720881 10.1038/s41598-017-05668-4 PMC5515942

[97] Markiewicz-Łoskot G, Kolarczyk E, Mazurek B, Łoskot M, Szydłowski L. Prolongation of electrocardiographic T wave parameters recorded during the head-up tilt table test as independent markers of syncope severity in children. Int J Environ Res Public Health. 2020;17:6441. 32899625 10.3390/ijerph17186441 PMC7558512

[98] Chu WH, Wu LJ, Wang C, Lin P, Li F, Zhu LP, et al. Evaluation of psychological fear in children undergoing head-up tilt test. Zhongguo Dang Dai Er Ke Za Zhi. 2014;16:263–7 (**in Chinese**). 24661518

[99] Lai WT, Chen MR, Lin SM, Hwang H. Application of head-up tilt table testing in children. J Formos Med Assoc. 2010;109:641–6. 20863991 10.1016/S0929-6646(10)60104-0

[100] Wang S, Peng Y, Wang Y, Li F, Xu Y, Zheng H, et al. Relationship between syncopal symptoms and HUTT modes. Cardiol Young. 2024. 10.1017/S1047951124000726. 38577783 10.1017/S1047951124000726

[101] Cui YX, Du JB, Zhang QY, Liao Y, Liu P, Wang YL, et al. A 10-year retrospective analysis of spectrums and treatment options of orthostatic intolerance and sitting intolerance in children. Beijing Da Xue Xue Bao Yi Xue Ban. 2022;54:954–60 (**in Chinese**). 36241239 10.19723/j.issn.1671-167X.2022.05.024 PMC9568388

[102] Gao Y, Jin H, Du J. Sitting intolerance: a new disease entity in children and adolescents. Pediatr Investig. 2022;6:299–301. 36582277 10.1002/ped4.12352 PMC9789932

[103] Cai H, Wang S, Zou R, Liu P, Li F, Wang Y, et al. Comparison of the active sitting test and head-up tilt test for diagnosis of postural tachycardia syndrome in children and adolescents. Front Pediatr. 2021;9:691390. 34604136 10.3389/fped.2021.691390 PMC8485704

[104] Boris JR, Huang J, Shuey T, Bernadzikowski T, Boris JR, Huang J, et al. Family history of associated disorders in patients with postural tachycardia syndrome. Cardiol Young. 2020;30:388–94. 32008600 10.1017/S1047951120000165

[105] Landwehr K, Meyer S, Flotats-Bastardas M, Poryo M. Syncope in children and adolescents are the current guidelines being followed? Wien Med Wochenschr. 2021;171:157–64 (**in German**). 33439378 10.1007/s10354-020-00798-3 PMC8057999

[106] Blanc JJ. Clinical laboratory testing: what is the role of tilt-table testing, active standing test, carotid massage, electrophysiological testing and ATP test in the syncope evaluation? Prog Cardiovasc Dis. 2013;55:418–24. 23472780 10.1016/j.pcad.2012.11.002

[107] Stewart JM, van Dijk JG, Balaji S, Sutton R. A framework to simplify paediatric syncope diagnosis. Eur J Pediatr. 2023;182:4771–80. 37470792 10.1007/s00431-023-05114-w PMC10640507

[108] Wang C, Mao D, Li M, Lin P, Li W, Zheng H. Diagnostic difference of head-up tilt table test in the patients of unexplained dizziness and syncope. Chin J Crit Care Med. 2005;25:796–9 (**in Chinese**).

[109] Wang Y, Wang S, Zou R, Chen S, Li F, Wang Y, et al. The relationship between unexplained chest pain in children and head-up tilt test. Front Pediatr. 2022;10:901919. 35722475 10.3389/fped.2022.901919 PMC9203148

[110] Zou R, Wang S, Li F, Lin P, Zhang J, Wang Y, et al. Clinical characteristics and hemodynamic responses to head-up tilt test in children and adolescents with unexplained sighing. Neurol Sci. 2021;42:3343–7. 33411193 10.1007/s10072-020-04956-8

[111] Gan T, Wu L, Zou R, Lin P, Li F, Yang H, et al. Relationship between unexplained palpitation in children and head-up tilt test. Zhong Nan Da Xue Xue Bao Yi Xue Ban. 2018;43:282–6 (**in Chinese**). 29701190 10.11817/j.issn.1672-7347.2018.03.008

[112] Yan H, Zhang C, Du J, Jin H. Clinical significance of abdominal pain and other gastrointestinal manifestations in children with autonomic nervous-mediated syncope. Chin J Pract Pediatr. 2013;28:43–7 (**in Chinese**).

[113] Zhang LN, Moak JP, Desbiens J, Hanumanthaiah S, Fabian RR, Clarke L, et al. Utility of diagnostic studies for upper gastrointestinal symptoms in children with orthostatic intolerance. J Pediatr. 2019;205:138–44. 30529135 10.1016/j.jpeds.2018.09.048

[114] Yang J, Li H, Ochs T, Zhao J, Zhang Q, Du S, et al. Erythrocytic hydrogen sulfide production is increased in children with vasovagal syncope. J Pediatr. 2015;166:965–9. 25641243 10.1016/j.jpeds.2014.12.021

[115] Zhang QY, Du JB, Zhen JL, Li WZ, Wang YL. Hemodynamic changes during head-up tilt test and predictive value thereof in predicting the efficacy of metoprolol therapy in children with vasovagal syncope. Zhonghua Yi Xue Za Zhi. 2007;87:1260–2 (**in Chinese**). 17686261

[116] Lin J, Han Z, Li X, Ochs T, Zhao J, Zhang X, et al. Risk factors for postural tachycardia syndrome in children and adolescents. PLoS One. 2014;9:e113625. 25474569 10.1371/journal.pone.0113625 PMC4256207

[117] Zhang Q, Chen X, Li J, Du J. Clinical features of hyperadrenergic postural tachycardia syndrome in children. Pediatr Int. 2014;56:813–6. 24862636 10.1111/ped.12392

[118] Hu Y, He B, Han Z, Wang Y, Tao C, Wang Y, et al. Risk factors for orthostatic hypertension in children. J Pediatr. 2020;227:212–7.e1. 32668285 10.1016/j.jpeds.2020.07.030

[119] Zhao J, Yang JY, Jin HF, Du JB. Clinical analysis of orthostatic hypertension in children. Zhonghua Er Ke Za Zhi. 2012;50:839–42 (**in Chinese**). 23302615

[120] Tao C, Cui Y, Zhang C, Liu X, Zhang Q, Liu P, et al. Clinical efficacy of empirical therapy in children with vasovagal syncope. Children (Basel). 2022;9:1065. 35884049 10.3390/children9071065 PMC9315970

[121] Xiao YY, Jin M, Ye WQ, Han L, Jin HF. Individualized treatment of syncope in children: state-of-the-art. Chin Med J (Engl). 2017;130:2878–80. 29176147 10.4103/0366-6999.219154 PMC5717869

[122] Xu WR, Du JB, Jin HF. Can pediatric vasovagal syncope be individually managed? World J Pediatr. 2022;18:4–6. 34982400 10.1007/s12519-021-00495-0

[123] Zhao T, Wang S, Wang M, Cai H, Wang Y, Xu Y, et al. Research progress on the predictive value of electrocardiographic indicators in the diagnosis and prognosis of children with vasovagal syncope. Front Cardiovasc Med. 2022;9:916770. 35935631 10.3389/fcvm.2022.916770 PMC9353577

[124] Yan H, Wang S, Cai H, Zhang J, Liu P, Wang Y, et al. Prognostic value of biomarkers in children and adolescents with orthostatic intolerance. Front Pediatr. 2021;9:752123. 34888267 10.3389/fped.2021.752123 PMC8650092

[125] Cheng W, Wang J, Lin J. Biomarkers and hemodynamic parameters in the diagnosis and treatment of children with postural tachycardia syndrome and vasovagal syncope. Int J Environ Res Public Health. 2022;19:6974. 35742222 10.3390/ijerph19126974 PMC9222341

[126] McIntyre-Patton L, Wanderski S, Graef D, Woessner L, Baker R. Randomized trial evaluating the effectiveness of a leg crossing and muscle tensing technique on decreasing vasovagal symptoms among pediatric and young adult patients undergoing peripheral IV catheter insertion. J Pediatr Nurs. 2018;38:53–6. 29167081 10.1016/j.pedn.2017.09.012

[127] Liu P, Mei W, Zhou M, Zhao T, Wang Y, Zou R, et al. Application of mind map can promote the health education effect of children with vasovagal syncope. Front Cardiovasc Med. 2023;10:1051677. 36873412 10.3389/fcvm.2023.1051677 PMC9978210

[128] Julliand S, Desmarest M, Gonzalez L, Ballestero Y, Martinez A, Moretti R, et al. Recovery position significantly associated with a reduced admission rate of children with loss of consciousness. Arch Dis Child. 2016;101:521–6. 26811367 10.1136/archdischild-2015-308857

[129] Williams EL, Khan FM, Claydon VE. Counter pressure maneuvers for syncope prevention: a semi-systematic review and meta-analysis. Front Cardiovasc Med. 2022;9:1016420. 36312294 10.3389/fcvm.2022.1016420 PMC9606335

[130] Wieling W, van Dijk N, Thijs RD, de Lange FJ, Krediet CT, Halliwill JR. Physical countermeasures to increase orthostatic tolerance. J Intern Med. 2015;277:69–82. 24697914 10.1111/joim.12249

[131] Jensen JL, Ohshimo S, Cassan P, Meyran D, Greene J, Ng KC, et al. Immediate interventions for presyncope of vasovagal or orthostatic origin: a systematic review. Prehosp Emerg Care. 2020;24:64–76. 30957664 10.1080/10903127.2019.1605431

[132] Anderson JB, Czosek RJ, Knilans TK, Marino BS. The effect of pediatric syncope on health-related quality of life. Cardiol Young. 2012;22:583–8. 22348920 10.1017/S1047951112000133

[133] Zhang Q, Jin H, Wang L, Chen J, Tang C, Du J. Randomized comparison of metoprolol versus conventional treatment in preventing recurrence of vasovagal syncope in children and adolescents. Med Sci Monit. 2008;14:CR199–203 18376348

[134] Tao C, Li X, Tang C, Jin H, Du J. Acceleration index predicts efficacy of orthostatic training on vasovagal syncope in children. J Pediatr. 2019;207:54–8. 30528576 10.1016/j.jpeds.2018.10.063

[135] Tao CY, Tang CS, Chen S, Jin HF, Du JB. Autonomic nervous function in vasovagal syncope of children and adolescents. Neurosci Bull. 2019;35:937–40. 31030406 10.1007/s12264-019-00383-8 PMC6754486

[136] Chun KJ, Yim HR, Park J, Park SJ, Park KM, On YK, et al. Role of baroreflex sensitivity in predicting tilt training response in patients with neurally mediated syncope. Yonsei Med J. 2016;57:313–20. 26847281 10.3349/ymj.2016.57.2.313 PMC4740521

[137] Gu S, Wang S, Wang Y, Liu P, Zhang J, Cai H, et al. Prognosis value of blood pressure variability in children with vasoinhibitory type vasovagal syncope. Indian J Pediatr. 2023;90:409–10. 36753018 10.1007/s12098-023-04505-z

[138] Chang L, Peng L, Liu J, Wang M, Li M, Kong Q, et al. Predictive analysis of catecholamines and electrolytes for recurrence of orthostatic intolerance in children. Front Pediatr. 2023;11:1220990. 37705599 10.3389/fped.2023.1220990 PMC10495584

[139] Subspecialty Group of Cardiology, Chinese Medical Association; Society of Pediatrics, Chinese Medical Association; Editorial Board, Chinese Journal of Pediatrics; Subspecialty Group of Cardiology, Beijing Medical Association; Society of Pediatrics, Beijing Medical Association. Expert consensus on the treatment of vasovagal syncope and postural tachycardia syndrome in children. Zhonghua Er Ke Za Zhi. 2018;56:6–9 (**in Chinese**). 10.3760/cma.j.issn.0578-1310.2018.01.003 29342989

[140] Song J, Tao C, Chen G, Chen S, Xu W, Du J, et al. Reduced 24-h sodium excretion is associated with a disturbed plasma acylcarnitine profile in vasovagal syncope children: a pilot study. Front Pediatr. 2020;8:98. 32219086 10.3389/fped.2020.00098 PMC7078237

[141] Wen C, Wang S, Zou R, Wang Y, Tan C, Xu Y, et al. Duration of treatment with oral rehydration salts for vasovagal syncope in children and adolescents. Turk J Pediatr. 2020;62:820–5. 33108085 10.24953/turkjped.2020.05.014

[142] Wang Y, Wang Y, Li X, Du J, Zhang H, Jin H, et al. Efficacy of increased salt and water intake on pediatric vasovagal syncope: a meta-analysis based on global published data. Front Pediatr. 2021;9:663016. 34055695 10.3389/fped.2021.663016 PMC8155624

[143] Du X, Tao C, Li X, Du J, Liao Y, Jin H. Predicting therapeutic efficacy of oral rehydration salts in children with vasovagal syncope. Front Pediatr. 2023;11:1164304. 37124188 10.3389/fped.2023.1164304 PMC10133722

[144] Tao CY, Chen S, Li XY, Tang CS, Du JB, Jin HF. Body mass index is a promising predictor of response to oral rehydration saline in children with vasovagal syncope. Chin Med J (Engl). 2020;134:463–8. 33617185 10.1097/CM9.0000000000001168 PMC7909309

[145] Du X, Tao C, Wang Y, Sun Y, Zhang Q, Zhang C, et al. Twenty-four-hour urinary sodium excretion predicts therapeutic effectiveness of oral rehydration saline in pediatric vasovagal syncope. Children (Basel). 2022;9:992. 35883976 10.3390/children9070992 PMC9321383

[146] Chu W, Wang C, Wu L, Lin P, Li F, Zou R. Oral rehydration salts: an effective choice for the treatment of children with vasovagal syncope. Pediatr Cardiol. 2015;36:867–72. 25577227 10.1007/s00246-015-1097-5

[147] Li W, Wang S, Liu X, Zou R, Tan C, Wang C. Assessment of efficacy of oral rehydration salts in children with neurally mediated syncope of different hemodynamic patterns. J Child Neurol. 2019;34:5–10. 30324839 10.1177/0883073818803035

[148] Anderson JB, Willis M, Lancaster H, Leonard K, Thomas C. The evaluation and management of pediatric syncope. Pediatr Neurol. 2016;55:6–13. 26706050 10.1016/j.pediatrneurol.2015.10.018

[149] Zhang QY, Du JB, Tang CS. The efficacy of midodrine hydrochloride in the treatment of children with vasovagal syncope. J Pediatr. 2006;149:777–80. 17137891 10.1016/j.jpeds.2006.07.031

[150] Zhang F, Liao Y, Li X, Chen L, Jin H, Du J. The predictive value of flow-mediated vasodilation on therapeutic eficacy of midorine hydrochloride for vasovagal syncope in children. Chin J Pract Pediatr. 2012;27:102–5 (**in Chinese**).

[151] Li L, Zhao H, Ma X, Jiao F, Lin J. Calcitonin gene-related peptide predicts therapeutic response to midodrine hydrochloride in children with vasovagal syncope. Front Neurosci. 2022;16:1026539. 36267231 10.3389/fnins.2022.1026539 PMC9577468

[152] Du X, Li X, Zhang C, Liu P, Wang Y, Zhang Q, et al. Serum uric acid predicts therapeutic response to midodrine hydrochloride in children with vasovagal syncope: a pilot study. Eur J Pediatr. 2024;183:371–8. 37904034 10.1007/s00431-023-05297-2 PMC10858074

[153] Chen L, Du JB, Zhang QY, Wang C, Du ZD, Wang HW, et al. A multicenter study on treatment of autonomous nerve-mediated syncope in children with beta-receptor blocker. Zhonghua Er Ke Za Zhi. 2007;45:885–8 (**in Chinese**). 18339272

[154] Wang J, Liu X, Jin H, Du J. Markers for predicting the efficacy of beta-blockers in vasovagal syncope management in children: a mini-review. Front Cardiovasc Med. 2023;10:1131967. 36970341 10.3389/fcvm.2023.1131967 PMC10030864

[155] Kong Q, Yang X, Cai Z, Pan Y, Wang M, Liu M, et al. Twenty-four-hour urine NE level as a predictor of the therapeutic response to metoprolol in children with recurrent vasovagal syncope. Ir J Med Sci. 2019;188:1279–87. 30761458 10.1007/s11845-019-01979-9

[156] Liao Y, Li XY, Zhang YW, Du JB. Meta-analysis of beta-adrenoceptor blockers for the treatment of vasovagal syncope. Beijing Da Xue Xue Bao Yi Xue Ban. 2008;40:603–9 (**in Chinese**). 19088832

[157] Tao C, Li X, Tang C, Jin H, Du J. Baroreflex sensitivity predicts response to metoprolol in children with vasovagal syncope: a pilot study. Front Neurosci. 2019;13:1329. 31920498 10.3389/fnins.2019.01329 PMC6923178

[158] Cui YX, Du JB, Jin HF. Baroreflex sensitivity and its implication in neurally mediated syncope in children. World J Pediatr. 2023;19:1023–9. 37014537 10.1007/s12519-023-00693-y

[159] Song J, Li H, Wang Y, Liu P, Li X, Tang C, et al. Left ventricular ejection fraction and fractional shortening are useful for the prediction of the therapeutic response to metoprolol in children with vasovagal syncope. Pediatr Cardiol. 2018;39:1366–72. 29767293 10.1007/s00246-018-1904-x

[160] Yuan P, Li X, Tao C, Du X, Zhang C, Du J, et al. Poincaré plot can be a useful Tool to select potential responders to metoprolol therapy in children with vasovagal syncope. Int J Gen Med. 2022;15:2681–93. 35300141 10.2147/IJGM.S352928 PMC8922042

[161] Yi S, Kong YH, Kim SJ. Fludrocortisone in pediatric vasovagal syncope: a retrospective, single-center observational study. J Clin Neurol. 2021;17:46–51. 33480198 10.3988/jcn.2021.17.1.46 PMC7840327

[162] Lenk M, Alehan D, Ozme S, Celiker A, Ozer S. The role of serotonin re-uptake inhibitors in preventing recurrent unexplained childhood syncope—a preliminary report. Eur J Pediatr. 1997;156:747–50. 9365060 10.1007/s004310050704

[163] Akella K, Olshansky B, Lakkireddy D, Gopinathannair R. Pacing therapies for vasovagal syncope. J Atr Fibrillation. 2020;13:2406. 33024506 10.4022/jafib.2406 PMC7533132

[164] Morillo CA, Brignole M. Pacing for vasovagal syncope: tips for use in practice. Auton Neurosci. 2022;241:102998. 35696879 10.1016/j.autneu.2022.102998

[165] Paech C, Wagner F, Mensch S, Antonin GR. Cardiac pacing in cardioinhibitory syncope in children. Congenit Heart Dis. 2018;13:1064–8. 30298977 10.1111/chd.12682

[166] Xu L, Zhao Y, Duan Y, Wang R, Hou J, Wang J, et al. Clinical efficacy of catheter ablation in the treatment of vasovagal syncope. J Clin Med. 2022;11:5371. 36143017 10.3390/jcm11185371 PMC9501086

[167] Li H, Shao W, Yu X, Gao L, Yuan Y. Efficacy of catheter ablation in ganglionated plexus for malignant vasovagal syncope children. Cardiol Young. 2024. 10.1017/S1047951124000659. 38572563 10.1017/S1047951124000659

[168] Li J, Zhang Q, Hao H, Jin H, Du J. Clinical features and management of postural tachycardia syndrome in children: a single-center experience. Chin Med J (Engl). 2014;127:3684–9. 25382319

[169] Tao CY, Jin HF, Du JB. Management of orthostatic intolerance in children the state of the art. World J Pediatr. 2020;16:543–8. 31912316 10.1007/s12519-019-00329-0

[170] Zheng X, Chen Y, Du J. Recent advances in the understanding of the mechanisms underlying postural tachycardia syndrome in children: practical implications for treatment. Cardiol Young. 2017;27:413–7. 27938459 10.1017/S1047951116002559

[171] Bai W, Han Z, Chen S, Li H, Song J, Qi J, et al. Serum resistin negatively correlates with clinical severity of postural tachycardia syndrome in children. Pediatr Cardiol. 2017;38:1639–44. 28828503 10.1007/s00246-017-1708-4

[172] Bai W, Chen SY, Jin HF, Du JB. Vascular dysfunction of postural tachycardia syndrome in children. World J Pediatr. 2018;14:13–7. 29411325 10.1007/s12519-017-0104-8

[173] Cutitta KE, Self M, de la Uz C. Postural orthostatic tachycardia syndrome (POTS) in teens: a guide for behavior change to manage symptoms. Pac Clin Electrophysiol. 2019;42:283–6. 10.1111/pace.13571 30520065

[174] Lei LY, Chew DS, Sheldon RS, Raj SR. Evaluating and managing postural tachycardia syndrome. Cleve Clin J Med. 2019;86:333–44. 31066664 10.3949/ccjm.86a.18002

[175] Chen G, Du J, Jin H, Huang Y. Postural tachycardia syndrome in children and adolescents: pathophysiology and clinical management. Front Pediatr. 2020;8:474. 32974246 10.3389/fped.2020.00474 PMC7468430

[176] Xu WR, Jin HF, Du JB. Pathogenesis and individualized treatment for postural tachycardia syndrome in children. Chin Med J (Engl). 2016;129:2241–5. 27625098 10.4103/0366-6999.189915 PMC5022347

[177] Liao Y, Du J. Pathophysiology and individualized management of vasovagal syncope and postural tachycardia syndrome in children and adolescents: an update. Neurosci Bull. 2020;36:667–81. 32367250 10.1007/s12264-020-00497-4 PMC7271077

[178] Zhang Q, Xu B, Du J. Update of individualized treatment strategies for postural orthostatic tachycardia syndrome in children. Front Neurol. 2020;11:525. 32655482 10.3389/fneur.2020.00525 PMC7325969

[179] Lu W, Yan H, Wu S, Xu W, Jin H, Du J. Hemocytometric measures predict the efficacy of oral rehydration for children with postural tachycardia syndrome. J Pediatr. 2017;187:220–4. 28526222 10.1016/j.jpeds.2017.04.034

[180] Xu B, Gao Y, Zhang Q, Li X, Liu X, Du J, et al. Establishment and validation of a multivariate predictive model for the efficacy of oral rehydration salts in children with postural tachycardia syndrome. EBioMedicine. 2024;100:104951. 38171114 10.1016/j.ebiom.2023.104951 PMC10796963

[181] Wang Y, Du J, Li X, Liu P, Wang Y, Liao Y, Jin H. Impact of comorbidities on the prognosis of pediatric postural tachycardia syndrome. Int J Gen Med. 2021;14:8945–54. 34866935 10.2147/IJGM.S339805 PMC8636694

[182] Li J, Zhang Q, Liao Y, Zhang C, Hao H, Du J. The value of acetylcholine receptor antibody in children with postural tachycardia syndrome. Pediatr Cardiol. 2015;36:165–70. 25087056 10.1007/s00246-014-0981-8

[183] Lin J, Zhao H, Shen J, Jiao F. Salivary cortisol levels predict therapeutic response to a sleep-promoting method in children with postural tachycardia syndrome. J Pediatr. 2017;191:91–5.e1. 29037796 10.1016/j.jpeds.2017.08.039

[184] Bruce BK, Harrison TE, Bee SM, Luedtke CA, Porter CJ, Fischer PR, et al. Improvement in functioning and psychological distress in adolescents with postural orthostatic tachycardia syndrome. Clin Pediatr (Phila). 2016;55:1300–4. 26983448 10.1177/0009922816638663

[185] Lu W, Yan H, Wu S, Chen S, Xu W, Jin H, et al. Electrocardiography-derived predictors for therapeutic response to treatment in children with postural tachycardia syndrome. J Pediatr. 2016;176:128–33. 27318378 10.1016/j.jpeds.2016.05.030

[186] Medow MS, Guber K, Chokshi S, Terilli C, Visintainer P, Stewart JM. The benefits of oral rehydration on orthostatic intolerance in children with postural tachycardia syndrome. J Pediatr. 2019;214:96–102. 31405524 10.1016/j.jpeds.2019.07.041 PMC6815702

[187] Zhang Q, Liao Y, Tang C, Du J, Jin H. Twenty-four-hour urinary sodium excretion and postural orthostatic tachycardia syndrome. J Pediatr. 2012;161:281–4. 22424949 10.1016/j.jpeds.2012.01.054

[188] Li H, Wang Y, Liu P, Chen Y, Feng X, Tang C, et al. Body mass index (BMI) is associated with the therapeutic response to oral rehydration solution in children with postural tachycardia syndrome. Pediatr Cardiol. 2016;37:1313–8. 27350278 10.1007/s00246-016-1436-1

[189] Lin J, Liu P, Wang Y, Li H, Li X, Zhao J, et al. Evaluation of the changes in heart rate during head-up test predicting the efficacy of oral rehydration salts on postural tachycardia syndrome in children. Zhonghua Er Ke Za Zhi. 2015;53:25–9 (**in Chinese**). 25748400

[190] Li H, Liao Y, Wang Y, Liu P, Sun C, Chen Y, et al. Baroreflex sensitivity predicts short-term outcome of postural tachycardia syndrome in children. PLoS One. 2016;11:e0167525. 27936059 10.1371/journal.pone.0167525 PMC5147897

[191] Chen L, Wang L, Sun J, Qin J, Tang C, Jin H, et al. Midodrine hydrochloride is effective in the treatment of children with postural orthostatic tachycardia syndrome. Circ J. 2011;75:927–31. 21301135 10.1253/circj.cj-10-0514

[192] Zhao J, Tang C, Jin H, Du J. Plasma copeptin and therapeutic effectiveness of midodrine hydrochloride on postural tachycardia syndrome in children. J Pediatr. 2014;165:290–4.e1. 24857518 10.1016/j.jpeds.2014.04.032

[193] Deng WJ, Liu YL, Liu AD, Holmberg L, Ochs T, Li X, et al. Difference between supine and upright blood pressure associates to the efficacy of midodrine on postural orthostatic tachycardia syndrome (POTS) in children. Pediatr Cardiol. 2014;35:719–25. 24253613 10.1007/s00246-013-0843-9

[194] Fan S, Cui Y, Liao Y, Jin H. Predicting therapeutic efficacy of pharmacological treatments in children with postural orthostatic tachycardia syndrome: a mini-review. Children (Basel). 2023;10:1093. 37508589 10.3390/children10071093 PMC10377884

[195] Lin J, Jin H, Du J. Assessment of therapeutic biomarkers in the treatment of children with postural tachycardia syndrome and vasovagal syncope. Cardiol Young. 2014;24:792–6. 24774832 10.1017/S1047951114000316

[196] Liao Y, Yang J, Zhang F, Chen S, Liu X, Zhang Q, et al. Flow-mediated vasodilation as a predictor of therapeutic response to midodrine hydrochloride in children with postural orthostatic tachycardia syndrome. Am J Cardiol. 2013;112:816–20. 23735645 10.1016/j.amjcard.2013.05.008

[197] Yang J, Zhao J, Du S, Liu D, Fu C, Li X, et al. Postural orthostatic tachycardia syndrome with increased erythrocytic hydrogen sulfide and response to midodrine hydrochloride. J Pediatr. 2013;163:1169–73.e2. 23726544 10.1016/j.jpeds.2013.04.039

[198] Li HX, Zheng XC, Chen SY, Liao Y, Han ZH, Huang P, et al. Increased endogenous sulfur dioxide involved in the pathogenesis of postural tachycardia syndrome in children. Chin Med J (Engl). 2018;131:435–9. 29451148 10.4103/0366-6999.225051 PMC5830828

[199] Zhang FW, Li XY, Ochs T, Chen L, Liao Y, Tang C, et al. Midregional pro-adrenomedullin as a predictor for therapeutic response to midodrine hydrochloride in children with postural orthostatic tachycardia syndrome. J Am Coll Cardiol. 2012;60:315–20. 22813609 10.1016/j.jacc.2012.04.025

[200] Yuan P, Lian Z, Wang Y, Zhang C, Jin H, Du J, et al. Poincaré plot can help predict the curative effect of metoprolol for pediatric postural orthostatic tachycardia syndrome. Front Neurosci. 2023;17:1280172. 38033543 10.3389/fnins.2023.1280172 PMC10682374

[201] Yang J, Liao Y, Zhang F, Chen L, Du J, Jin H. The follow-up study on the treatment of children with postural orthostatic tachycardia syndrome. Int J Pediatr. 2014;41:76–9.

[202] Deng W, Yang J, Liu Y, Du J, Jin H. A time-effect analysis of postural orthostatic tachycardia syndrome treated with midodrine hydrochloride in children. Chin J Pract Pediatr. 2013;28:274–6 (**in Chinese**).

[203] Zhao J, Du S, Yang J, Lin J, Tang C, Du J, et al. Usefulness of plasma copeptin as a biomarker to predict the therapeutic effectiveness of metoprolol for postural tachycardia syndrome in children. Am J Cardiol. 2014;114:601–5. 24996552 10.1016/j.amjcard.2014.05.039

[204] Lin J, Han Z, Li H, Chen SY, Li X, Liu P, et al. Plasma C-type natriuretic peptide as a predictor for therapeutic response to metoprolol in children with postural tachycardia syndrome. PLoS One. 2015;10:e0121913. 25811760 10.1371/journal.pone.0121913 PMC4374798

[205] Zhang Q, Chen X, Li J, Du J. Orthostatic plasma norepinephrine level as a predictor for therapeutic response to metoprolol in children with postural tachycardia syndrome. J Transl Med. 2014;12:249. 25204388 10.1186/s12967-014-0249-3 PMC4177336

[206] Cui Y, Wang Y, Liu P, Wang Y, Du J, Jin H. Baroreflex sensitivity predicts therapeutic effects of metoprolol on pediatric postural orthostatic tachycardia syndrome. Front Cardiovasc Med. 2022;9:930994. 36187012 10.3389/fcvm.2022.930994 PMC9515359

[207] Wang Y, Zhang C, Chen S, Liu P, Wang Y, Tang C, et al. Heart rate variability predicts therapeutic response to metoprolol in children with postural tachycardia syndrome. Front Neurosci. 2019;13:1214. 31780890 10.3389/fnins.2019.01214 PMC6861190

[208] Wang Y, Sun Y, Zhang Q, Zhang C, Liu P, Wang Y, et al. Baseline corrected QT interval dispersion is useful to predict effectiveness of metoprolol on pediatric postural tachycardia syndrome. Front Cardiovasc Med. 2022;8:808512. 35127870 10.3389/fcvm.2021.808512 PMC8812810

[209] Xu BW, Zhang QY, Li XY, Tang CS, Du JB, Liu XQ, et al. A predictive model of response to metoprolol in children and adolescents with postural tachycardia syndrome. World J Pediatr. 2023;19:390–400. 36781629 10.1007/s12519-022-00677-4 PMC10060270

[210] Wang S, Zou R, Cai H, Wang Y, Ding Y, Tan C, et al. Heart rate and heart rate difference predicted the efficacy of metoprolol on postural tachycardia syndrome in children and adolescents. J Pediatr. 2020;224:110–4. 32464225 10.1016/j.jpeds.2020.05.017

[211] Wang S, Zou R, Cai H, Wang C. Prognostic value of heart rate and blood pressure on the prognosis of postural tachycardia syndrome in children. Front Pediatr. 2022;10:802469. 35433537 10.3389/fped.2022.802469 PMC9005773

[212] Deng X, Zhang Y, Liao Y, Du J. Efficacy of β-blockers on postural tachycardia syndrome in children and adolescents: a systematic review and meta-analysis. Front Pediatr. 2019;7:460. 31788462 10.3389/fped.2019.00460 PMC6854016

[213] Guven B, Oner T, Tavli V, Yilmazer MM, Demirpence S, Mese T. Low iron storage in children with tilt positive neurally mediated syncope. World J Pediatr. 2013;9:146–51. 23275108 10.1007/s12519-012-0396-7

[214] Blitshteyn S. Vitamin B1 deficiency in patients with postural tachycardia syndrome (POTS). Neurol Res. 2017;39:685–8. 28531358 10.1080/01616412.2017.1331895

[215] Do T, Diamond S, Green C, Warren M. Nutritional implications of patients with dysautonomia and hypermobility syndromes. Curr Nutr Rep. 2021;10:324–33. 34510391 10.1007/s13668-021-00373-1 PMC8435108

[216] Raj SR, Fedorowski A, Sheldon RS. Diagnosis and management of postural orthostatic tachycardia syndrome. CMAJ. 2022;194:E378–85. 35288409 10.1503/cmaj.211373 PMC8920526

[217] Gibbons CH, Schmidt P, Biaggioni I, Frazier-Mills C, Freeman R, Isaacson S, et al. The recommendations of a consensus panel for the screening, diagnosis, and treatment of neurogenic orthostatic hypotension and associated supine hypertension. J Neurol. 2017;264:1567–82. 28050656 10.1007/s00415-016-8375-x PMC5533816

[218] Loughlin EA, Judge CS, Gorey SE, Costello MM, Murphy RP, Waters RF, et al. Increased salt intake for orthostatic intolerance syndromes: a systematic review and meta-analysis. Am J Med. 2020;133:1471–8.e4. 32603788 10.1016/j.amjmed.2020.05.028

[219] Raj SR, Coffin ST. Medical therapy and physical maneuvers in the treatment of the vasovagal syncope and orthostatic hypotension. Prog Cardiovasc Dis. 2013;55:425–33. 23472781 10.1016/j.pcad.2012.11.004 PMC3594734

[220] Hu Y, Jin H, Du J. Orthostatic hypertension in children: an update. Front Pediatr. 2020;8:425. 32850540 10.3389/fped.2020.00425 PMC7403181

[221] Gu S, Wang S, Wang Y, Zhang J, Cai H, Zou R, et al. Changes of blood pressure variability in children with postural tachycardia syndrome. Children (Basel). 2023;10:1244. 37508741 10.3390/children10071244 PMC10378093

[222] Peng Y, Wang S, Zou R, Cai H, Zhang J, Wang Y, et al. The influence of sex on the treatment of postural tachycardia syndrome in children. Medicine (Baltimore). 2023;102:e33951. 37443510 10.1097/MD.0000000000033951 PMC10344500

[223] Li Y, Li H, Li X, Li X, Jin H. Prognostic analysis of orthostatic intolerance using survival model in children. Chin Med J (Engl). 2014;127:3690–4. 25382320

[224] Tao C, Xu B, Liao Y, Li X, Jin H, Du J. Predictor of syncopal recurrence in children with vasovagal syncope treated with metoprolol. Front Pediatr. 2022;10:870939. 35463909 10.3389/fped.2022.870939 PMC9024146

[225] Sun R, Kang Y, Zhang M, Wang H, Shi L, Li X. Development of prognostic nomogram model to predict syncope recurrence in children with vasovagal syncope. Front Cardiovasc Med. 2023;10:1099115. 36970348 10.3389/fcvm.2023.1099115 PMC10031040

[226] Sun R, Kang Y, Zhang M, Wang H, Shi L, Li X. Development of a nomogram model to predict malignant vasovagal syncope in Chinese children. Front Pediatr. 2023;11:1158537. 37077332 10.3389/fped.2023.1158537 PMC10109463

[227] Song JY, Li HX, Li XY, Wang YL, Jin HF, Du JB. Relationship between blood routine test parameters and syncopal recurrence of vasovagal syncope in children. Zhonghua Er Ke Za Zhi. 2017;55:59–63 (**in Chinese**). 28072962 10.3760/cma.j.issn.0578-1310.2017.01.012

[228] Tao C, Lu W, Lin J, Li H, Li X, Tang C, et al. Long-term outcomes of children and adolescents with postural tachycardia syndrome after conventional treatment. Front Pediatr. 2019;7:261. 31316954 10.3389/fped.2019.00261 PMC6610301

[229] Li H, Zhang F, Wang Y, Liu P, Zhang C, Feng X, et al. Predictive value of baseline plasma midregional fragment of pro-adrenomedullin level on long-term outcome of postural tachycardia syndrome children treated with midodrine hydrochloride. Zhonghua Xin Xue Guan Bing Za Zhi. 2015;43:507–10 (**in Chinese**). 26420118

[230] Bhatia R, Kizilbash SJ, Ahrens SP, Killian JM, Kimmes SA, Knoebel EE, et al. Outcomes of adolescent-onset postural orthostatic tachycardia syndrome. J Pediatr. 2016;173:149–53. 26979650 10.1016/j.jpeds.2016.02.035

